# Physiological, biochemical and molecular signaling basis of cold stress tolerance in plants

**DOI:** 10.3389/fpls.2025.1707204

**Published:** 2025-12-19

**Authors:** Rajib Roychowdhury, Soumya Prakash Das, Puja Sarkar, Zeba Khan, Ajay Kumar, Umakanta Sarker, Radha Sivarajan Sajeevan

**Affiliations:** 1 Agricultural Research Organization (ARO) – Volcani Center, Rishon Lezion, Israel; 2 School of Life Sciences, Seacom Skills University, Bolpur, West Bengal, India; 3 Department of Genetics and Plant Breeding, Uttar Banga Krishi Viswavidyalaya, Cooch Behar, West Bengal, India; 4 Department of Botany, Raja Mahendra Pratap Singh State University, Aligarh, Uttar Pradesh, India; 5 Amity Institute of Biotechnology, Amity University, Noida, Uttar Pradesh, India; 6 Department of Genetics and Plant Breeding, Faculty of Agriculture, Gazipur Agricultural University, Gazipur, Bangladesh; 7 Department of Plant Protection Biology, Swedish University of Agricultural Sciences, Lomma, Sweden

**Keywords:** climate change, cold stress, crop improvement, low temperature, signaling

## Abstract

Cold stress significantly hampers plant growth, development, and yield, posing a threat to global food security. This review consolidates our understanding of the physiological, biochemical, and molecular mechanisms that enable plants to tolerate cold stress. Plants employ many strategies to mitigate the negative effects of cold, including osmotic adjustments, boosting antioxidant defences, accumulating osmoprotectants, and regulating cold-responsive genes via transcription factors such as C-repeat binding proteins. The CBF expression-1 C-repeat binding factors cold-regulated (ICE1-CBF-COR) genetic signalling pathway is vital for acclimatisation to low temperatures and boosting cold resistance. Understanding these systems is essential for producing crops capable of thriving in cold environments through breeding and biotechnology. Enhancing crop resistance to cold stress can promote sustainable agriculture and bolster food security amid climate change. This review highlights key findings, methodological limitations, and areas needing further research to support the development of cold-tolerant crop varieties in the face of climate change.

## 1 Introduction

Cold stress is a major environmental factor that substantially hampers plant growth, development, and geographical distribution, ultimately threatening global food security (Roychowdhury et al., 2020). Plants respond to cold stress by activating a series of physiological, biochemical, and molecular mechanisms to detect, adapt to, and counteract the damaging effects of low temperatures. Over time, plants have developed complex and interconnected strategies to cope with cold stress. These include osmotic adjustments through the accumulation of osmolytes such as proline and sugars, modifications in membrane lipid composition to preserve integrity, and altered water relations affecting stomatal conductance and water balance (Hasanuzzaman et al., 2015). In reaction to cold stress, plants biochemically strengthen their antioxidant defence systems to neutralise the excessive production of reactive oxygen species (ROS), which can cause oxidative damage. They produce osmoprotectants to shield cellular structures and macromolecules, and regulate the levels of phytohormones like abscisic acid (ABA), which are vital for stress signalling and response (Saha et al., 2025). At the molecular level, cold-regulated (COR) genes are activated, with transcription factors such as C-repeat binding factors (CBFs) playing essential roles in controlling gene expression that enhances plant survival under cold conditions. Furthermore, specific signalling pathways, notably the ICE1-CBF-COR pathway, are crucial for acclimatisation to cold and improving freezing tolerance (Feng et al., 2025a). Cold acclimatisation is a process that increases plant resistance to freezing temperatures following exposure to low, nonfreezing conditions. It involves several changes in gene expression, metabolism, and physiology. Understanding the physiological, biochemical, and molecular foundations of plant cold stress tolerance is vital for developing strategies to improve crop resistance to cold stress through breeding and biotechnology (Roychowdhury et al., 2023a). Enhancing crop resilience to cold temperatures can greatly benefit sustainable agriculture, particularly as climate change leads to greater temperature fluctuations, resulting in increased crop losses. Despite advancing our understanding of plant responses to cold stress, we remain insufficiently informed about the interaction of various systems governing cold tolerance and how this knowledge can be applied to increase crop resilience (Roychowdhury et al., 2023b). This review aims to synthesise current insights into the physiological, biochemical, and molecular pathways that enable cold stress tolerance in plants. It highlights key findings, analyses the regulatory pathways involved in cold stress responses, and suggests potential avenues for research that could contribute to the development of cold-tolerant crop varieties. Ultimately, this review seeks to promote food security by advancing resilient crops that can withstand climatic challenges, particularly cold stress, through elucidating the complex mechanisms underlying cold stress tolerance.

## 2 Physiological basis of cold stress tolerance in plants

Abiotic stress is a persistent phenomenon that causes changes in plant structure, functionality, chemistry, and molecular composition. These changes hinder plant growth and thereby reduce yield. Plants experience stress when exposed to extreme temperatures, either very high or very low. Cold stress is a major environmental factor that hampers plant growth and food production, especially in mountainous regions. Plants can tolerate cold stress by adapting and responding at the molecular, cellular, physiological, and biochemical levels (Feng et al., 2025a). Cold or low temperatures are among the most harmful factors that can affect plant growth and development. This stress can be classified as chilling stress (temperatures below 20°C) and freezing stress (temperatures below 0°C) (Levitt, 2012). Tropical and subtropical plants are sensitive to chilling stress because they generally grow in frost-free areas. Conversely, temperate plants may survive freezing conditions through a process called cold adaptation, which occurs after exposure to nonfreezing temperatures (Jahed et al., 2023). Short-term exposure to higher temperatures can increase plant tolerance to stress, a phenomenon known as acquired thermotolerance. Similarly, brief exposure to relatively low temperatures can enhance an individual's stress tolerance, known as chilling tolerance or cold acclimatisation. Food crops such as rice, maize, soybean, and sorghum are sensitive to temperature changes, and their normal growth and development are hindered below their optimal temperatures (Zhou et al., 2025). Temperature stress affects the energy usage of plants, impacts their cellular metabolism, and causes changes in the structure, function, and activity of enzymes and membrane metabolite transporters. Plant regulatory mechanisms aim to restore normal metabolite levels and metabolic fluxes (Satyakam et al., 2022). The metabolic changes during temperature stress are largely driven by evolutionary processes that increase crop tolerance. Several metabolites associated with the stress response are thought to have qualities that enhance plants’ ability to handle stress. Cold stress generally leads to several adverse effects, including poor seedling germination, leaf discolouration, wilting, and reduced tillering. During the reproductive phase, cold stress can inhibit flowering and cause pollen infertility (Huang et al., 2022). Many believe this is a major factor in declining grain yields. Cold stress is particularly damaging to plant plasma membranes. Crops, especially, can be cultivated more successfully in cold weather if they are resilient to frost during late spring and early autumn.

### 2.1 Plant response to cold stress

**Figure 1:**
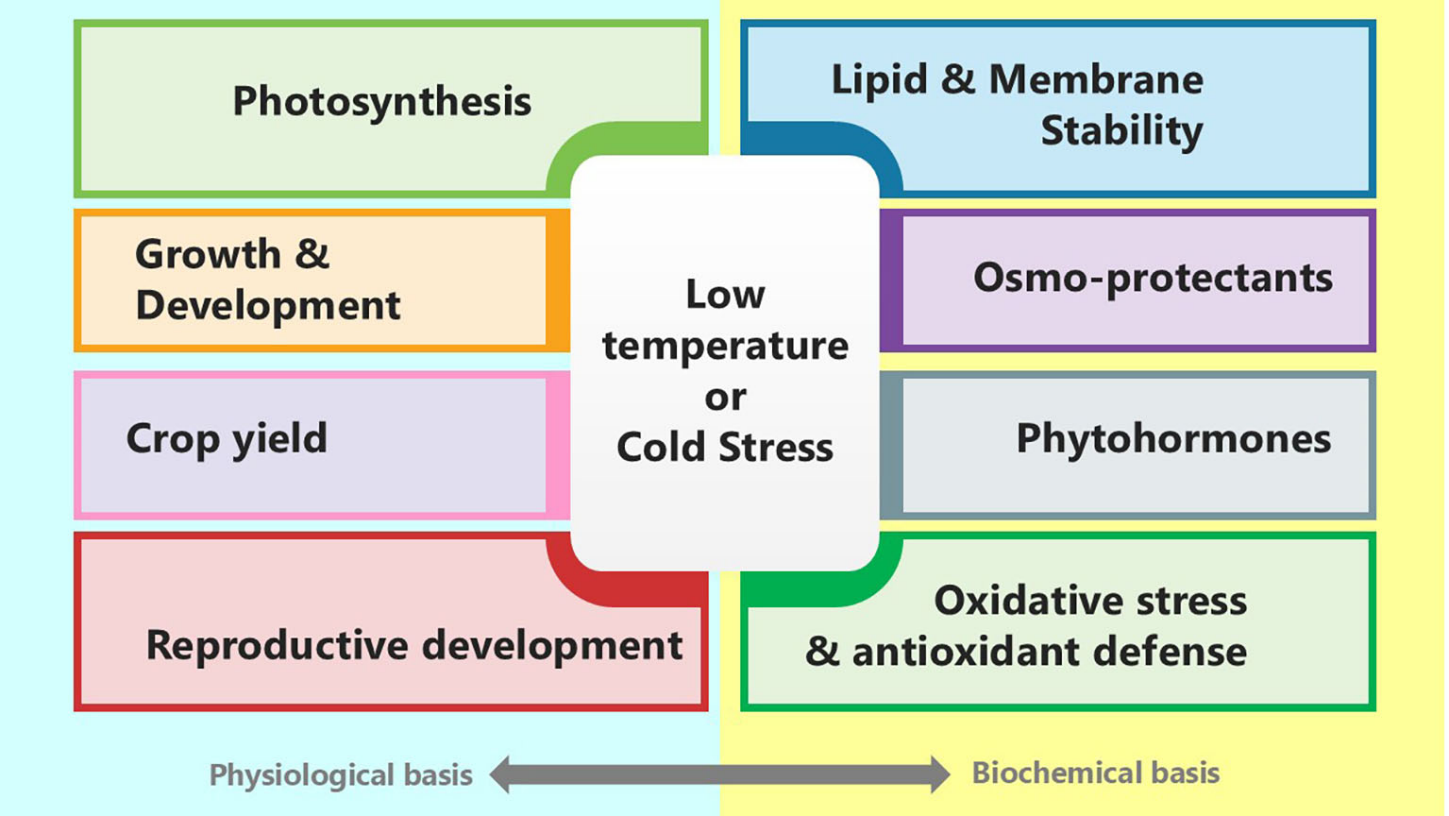
Diagram illustrating the effects of low temperature or cold stress on various physiological and biochemical aspects. Physiological effects include photosynthesis, growth and development, crop yield, and reproductive development. Biochemical effects include lipid and membrane stability, osmo-protectants, phytohormones, and oxidative stress and antioxidant defense.

Cold stress substantially affects all aspects of plant growth and production, including growth, photosynthesis, reproductive development, and ultimately yield (**Figure 1**). Plants respond to cold stress by undergoing various physiological, biochemical, and molecular changes that enhance their survival and reduce damage (Qian et al., 2024). Cold stress often hampers development by altering resource allocation and decreasing metabolic activity. Low temperatures impact photosynthesis, possibly reducing photosynthetic efficiency and causing photoinhibition. Reproductive development is especially vulnerable to cold stress, which can affect pollen viability, fertilisation, and seed set, ultimately influencing yield. Each plant requires an optimal temperature for proper growth and development. A temperature suitable for one plant may not be appropriate for another (Manasa et al., 2022). Therefore, understanding plant responses to cold stress in terms of growth, photosynthesis, reproductive development, and yield is vital for devising strategies to improve cold tolerance and sustain production in cold-prone regions.

Cold stress mainly affects the plasma membrane at both cellular and physiological levels, serving as the primary site for stress detection and damage. The plasma membrane consists of a lipid‒protein matrix, and the ratio of saturated to unsaturated fatty acids influences its flexibility and function (Cao et al., 2023). Saturated fatty acids solidify more quickly at low temperatures than unsaturated fatty acids, thus impacting membrane fluidity. When membranes are exposed to cold or freezing stress, they undergo a phase change from a semifluid to a semicrystalline state once the transition temperature (Tm) is reached. This change reduces permeability, alters the functioning of ion channels and transport proteins, and affects cellular signalling pathways. Cold stress also causes cells to lose water and electrolytes, significantly decreasing their chances of survival (Kim et al., 2024). The extent of this damage is heavily influenced by the composition of membrane lipids. Cold-sensitive plants typically have a higher proportion of saturated fatty acids, which raise the transition temperature and increase vulnerability to cold damage. Cold-tolerant plants contain more unsaturated fatty acids, which lower the transition temperature, maintain membrane fluidity under cold conditions, and support reproduction. Cold stress affects not only membrane dynamics but also hormonal regulation and metabolic processes essential for reproduction (Wu et al., 2022). This section explores the impact of cold stress on key aspects of plant development and productivity.

#### 2.1.1 Growth

Low temperatures are a major abiotic stress that presents a significant challenge during both seedling and reproductive stages. Common signs of cold stress in normal plant growth and development include chlorosis, loss of vigour, tissue damage, stunted seedlings, failure to germinate, and even necrosis (Theocharis et al., 2012). Cold stress causes increased membrane rigidity, disorganisation of the cytoskeleton, and decreased enzyme efficiency, making it harder to maintain cellular homeostasis. Freezing temperatures can even cause irreversible cellular damage and tissue necrosis. Extremely cold temperatures hinder plant growth and seed germination, leading to reduced food production. Cold stress during the reproductive stage causes notable yield loss in rice, resulting in increased spikelet sterility (Liu et al., 2019). During the grain-filling stage, which occurs between fertilisation and physiological maturity, the developing seed stores energy for germination, thereby affecting the crop's economic yield. Chilling stress can influence kernel development by altering cell division and differentiation, as well as affecting the rate and duration of grain filling. Cold stress impacts all aspects of cellular function in plants. Induced dehydration of membranes is a primary consequence of cold stress. Cold stress during the rice reproductive stage reportedly extends maturation time and reduces the number of panicles and spikelets (El-Refaee et al., 2024). Similarly, in maize, temperatures lower than optimum lead to decreased leaf area and up to a 50% reduction in shoot dry weight (Lainé et al., 2023). Research on another cold-sensitive crop, soybean, has revealed decreased chlorophyll fluorescence, impaired photosynthesis, and increased leaf injury (Hussain et al., 2023).

#### 2.1.2 Photosynthesis

Photosynthesis is one of the physiological systems most negatively impacted by cold stress. The chloroplast is the organelle most vulnerable to damage from chilling and freezing. Low temperatures compromise the integrity of thylakoid membranes, inhibit photosynthesis-related enzyme activity, and disrupt the balance among light absorption, electron transport, and carbon fixation (Banerjee and Roychoudhury, 2019). These problems make it harder for plants to absorb carbon and produce energy, thus hindering growth and reducing output potential. Plants naturally adapted to cold environments, such as alpine or boreal species, show structural and metabolic changes in their chloroplasts that enhance their tolerance and enable photosynthesis at low temperatures (Baker, 2019). Stomatal regulation in plants is among the first responses to cold stress. Stomata are tiny openings on leaf surfaces that control the exchange of CO₂ for photosynthesis and H₂O vapour during transpiration. Cold stress often causes stomata to close, reducing CO₂ availability to mesophyll cells and limiting the efficiency of the enzyme ribulose-1,5-bisphosphate carboxylase/oxygenase (RuBisCO) (Bhattacharya, 2022a). This stomatal limitation is linked to non-stomatal effects, such as decreased activity of Calvin cycle enzymes, lower ATP production, and altered chloroplast ultrastructure, all of which impair photosynthetic efficiency (Agurla et al., 2018). Stomatal density refers to the number of stomata per unit area of epidermis, while the stomatal index indicates the ratio of stomata to total epidermal cells. A significant correlation exists between stomatal density and photosynthetic capacity. Higher stomatal density allows more CO₂ to enter leaves, thus increasing the rate of photosynthesis under optimal conditions. Conversely, cold stress typically causes a reduction in stomatal conductance and changes in stomatal behaviour, which decrease the effectiveness of photosynthesis (Zhang et al., 2024a). Studies on various plant species show that environmental stresses, particularly extreme heat, can alter stomatal morphology and density. Wu et al. (2022) examined the effects of elevated temperatures on two Syzygium species and found that S. rehderianum experienced notable reductions in both stomatal size and density during heat stress, leading to decreased photosynthetic rates. In contrast, S. superbum maintained consistent stomatal size and density, thus keeping its photosynthetic capacity unaffected. This research highlights the species-specific variability in stomatal responses to environmental extremes, a theory also relevant for cold stress. Cold stress increases the risk of photoinhibition since low temperatures hinder plants’ ability to utilise the light energy they absorb. This causes an excessive slowdown in the photosynthetic electron transport chain and leads to the overproduction of reactive oxygen species (ROS). Oxidative stress damages the D1 protein of photosystem II (PSII), reduces chlorophyll content, and hampers the plant’s ability to absorb light (Adam and Murthy, 2013). Cold-tolerant plants mitigate this damage by boosting the xanthophyll cycle, enhancing antioxidant defences, and lowering lipid saturation in thylakoid membranes—actions that help maintain chloroplast function and stabilise photosynthesis (Bhattacharya, 2022a). The problems with photosynthesis during cold conditions mainly arise from a combination of stomatal restrictions, chloroplast dysfunction, photoinhibition, and metabolic limitations. A thorough understanding of these physiological mechanisms is essential for developing crop varieties that can withstand low temperatures and sustain photosynthesis despite environmental fluctuations.

#### 2.1.3 Reproductive development

Reproductive development is a crucial stage in a plant's life cycle, and cold stress significantly impacts it. Exposure to low temperatures can hinder flower initiation, prevent flowering, and reduce fertility, primarily by disrupting pollen formation (Albertos et al., 2019). Cold stress leads to pollen sterility, decreased pollen viability, problems with anther dehiscence, and slower pollen tube growth. Collectively, these issues reduce the effectiveness of fertilisation and lower seed or grain production. This stage-specific sensitivity explains why brief periods of chilling or freezing can cause substantial decreases in the yields of cereals, legumes, and horticultural crops. Low temperatures may inhibit the production of gibberellins, which are vital for flower induction (Takeno, 2016). They can also alter levels of abscisic acid (ABA) and cytokinins, thereby affecting pollen and ovule development. Cold stress restricts carbohydrate transport to developing floral tissues, resulting in a deficiency of essential assimilates necessary for pollen viability and seed set in reproductive organs. The disruption of reproductive development under cold stress is a complex process involving membrane rigidification, metabolic imbalance, hormonal disturbances, and insufficient expression of stress-responsive genes (Sharma and Nayyar, 2016). Understanding these physiological mechanisms offers key insights for breeding and biotechnological approaches aimed at developing crop varieties with enhanced cold tolerance and stable reproductive performance under challenging climatic conditions.

#### 2.1.4 Yield

Low temperatures are a major abiotic factor affecting both crop yield and geographical distribution. A slight decrease in temperature, even without visible damage, can lead to up to a 50% reduction in yield. Cold stress restricts the variety of crops that can be grown and significantly lowers agricultural productivity, depending on the duration, timing, and severity of exposure during the growing season (Staniak et al., 2021). Additionally, exposure to cold or freezing conditions accelerates the build-up of reactive oxygen species (ROS), causing oxidative damage, lipid peroxidation, and protein denaturation. Metabolic changes impair critical processes such as photosynthesis, glucose metabolism, food intake, and reproductive development, resulting in decreased yields. Cold stress impacts crop growth both directly and indirectly. However, milder but prolonged chilling stress can cause delayed germination, poor seedling establishment, reduced flowering, suboptimal grain filling, and lower seed quality (Soualiou et al., 2022). Cold stress can also decrease marketable quality, even if overall yields are not entirely lost. This can influence factors like seed size, oil content, starch accumulation, and postharvest storage capacity. Cold stress is reported to cause substantial financial losses in global agriculture. It is estimated to account for over $2 billion in annual crop losses worldwide. In 1995, early autumn frosts in the United States caused significant damage to maize and soybeans, with losses exceeding $1 billion (Elmore and Doupnik, 1995). Cold stress reduces yields directly and increases crop vulnerability to pests and viruses, thereby worsening economic impacts (Newton et al., 2011). Due to climate change, the occurrence of cold stress is expected to rise even in typically warm regions. This highlights the urgent need for developing cold-tolerant cultivars through traditional breeding, molecular breeding, and advanced biotechnological techniques, such as genome editing, transcriptome profiling, and metabolomic approaches.

### 2.2 Plant adaptation to cold stress

**Figure 2:**
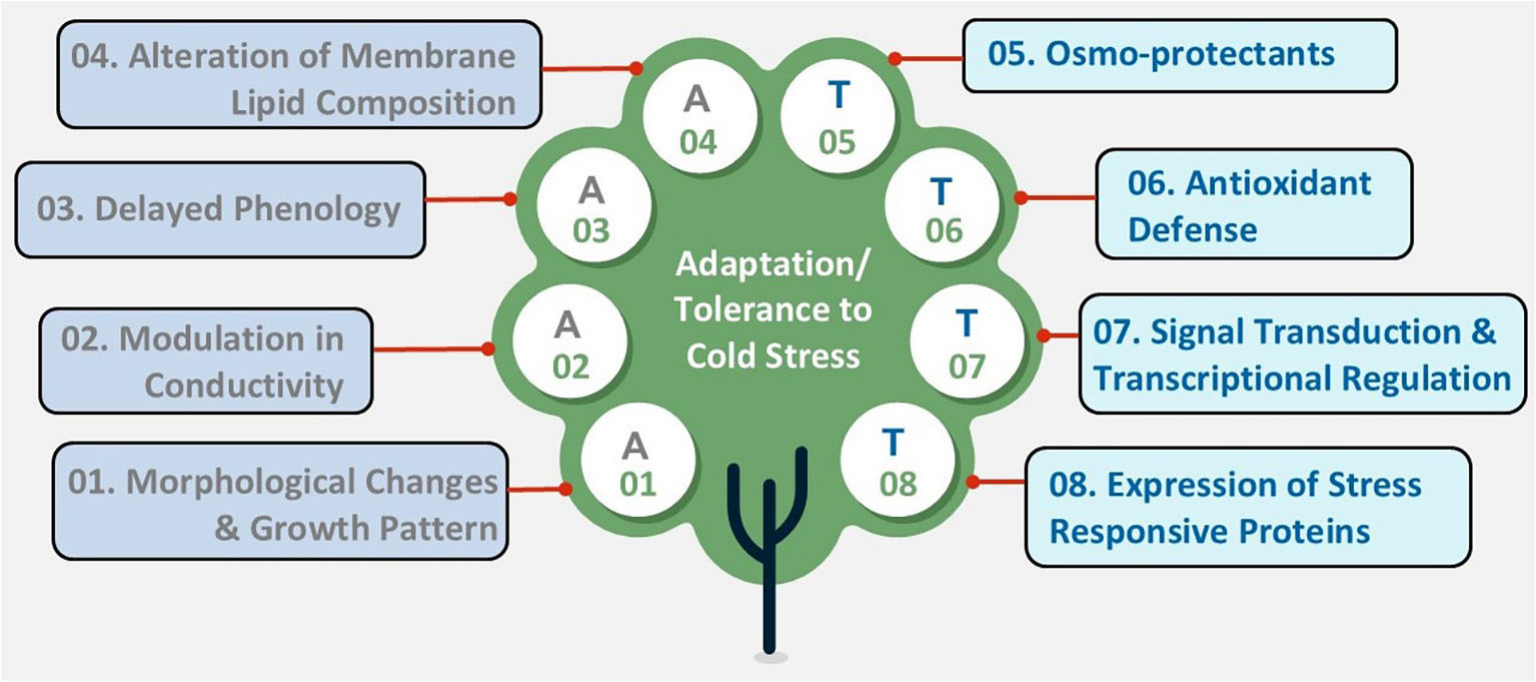
Diagram depicting strategies for adaptation (A) and tolerance (T) to cold stress, organized in a tree diagram. Nodes labeled A01 to A04 include morphological changes, modulation in conductivity, delayed phenology, and alteration of membrane lipid composition denotes adaptive strategies. Nodes labeled T05 to T08 include osmoprotectants, antioxidant defense, signal transduction and transcriptional regulation, and expression of stress-responsive proteins denotes tolerance mechanism.

Since the optimal temperature range differs among plants, each species responds uniquely to low temperatures. While crop plants such as rice, maize, soybean, and vegetables like tomatoes or potatoes are sensitive to chilling, crops such as oats can tolerate chilling but not freezing. Conversely, plants like wheat, barley, or rye can withstand freezing temperatures (Ding et al., 2019). Plants have developed various strategies to adapt to cold conditions, ensuring their survival and ongoing growth (Gusain et al., 2023). These adaptations generally fall into two main categories: avoidance mechanisms, which help plants prevent cold damage, and tolerance mechanisms, which enable plants to survive and operate under cold or freezing stress (**Figure 2**). The significance of these strategies varies among different plant species and ecosystems, reflecting the evolutionary pressures of their native environments.

#### 2.2.1 Cold avoidance mechanism

Plants employ avoidance techniques to decrease the risk of freezing or damaging their cells by modifying their physiological, anatomical, or phenological traits. While some of these modifications are reversible, others are permanent within the plants. The cold avoidance mechanism involves completing developmental stages either during the warmest periods or under prevailing cold conditions. Low temperatures are initially detected by the plasma membrane, and molecular changes induced by cold stress in the membrane composition help protect cells from damage. In plants such as Arabidopsis, alfalfa, and Brassica napus, the recognition of cold stress results in membrane rigidification followed by activation of downstream pathways (Ritonga and Chen, 2020). Supercooling is a well-studied mechanism through which plants avoid freezing (Reyes-Díaz et al., 2006). It occurs when water within plant tissues remains in a liquid state below its usual freezing point. This process is often seen in the xylem parenchyma cells of woody perennials like apple (Malus domestica), peach (Prunus persica), and birch (Betula spp.) (George and Burke, 1977). These plants survive subzero temperatures by preventing ice formation inside their cells, which would be lethal. Besides supercooling, plants adopt phenological avoidance strategies by adjusting their developmental cycles. Many temperate cereals, such as winter wheat and barley, reduce cold damage by entering a dormancy phase dependent on vernalisation (Hassan et al., 2021). Reproductive development only proceeds after an extended cold period, thereby lowering the risk of frost damage to flowers and seeds. Alpine flora like Ranunculus glacialis and Saxifraga oppositifolia complete their reproductive cycle during brief periods of favourable weather, avoiding prolonged exposure to freezing conditions (Klatt et al., 2018). Morphological adaptations also assist plants to retain warmth. For instance, compact rosette growth occurs in Arabidopsis thaliana, and cushion plants in alpine environments help conserve heat near the plant body, protecting tissues against sudden drops in temperature (Hoermiller et al., 2018). Evergreen conifers, such as Pinus and Picea species, have thin needle-like leaves with thick cuticles that prevent freezing of photosynthetic tissues and reduce transpiration (Wang et al., 2019).

#### 2.2.2 Cold tolerance mechanism

Cold tolerance refers to plants' ability to endure and survive in chilling (0 - 20°C) or freezing (<0°C) conditions through changes in their biochemistry, cellular structure, and molecular composition. Organisms often adapt to cold by altering membrane lipid composition, accumulating cryoprotective solutes, enhancing antioxidant defenses, and activating COR genes (Cheng et al., 2022). The physical state of the plasma membrane significantly influences the response of plants to low temperatures. A fluid membrane bilayer enables proper function of membrane proteins and maintains an effective permeability barrier. The ratio of unsaturated to saturated fatty acids in plasma membrane lipids determines membrane fluidity. Cold-sensitive plants' membranes are reported to contain a higher proportion of saturated fatty acids, while cold-tolerant plants generally have more unsaturated fatty acids. The primary response to cold stress involves the transition of membranes from a semifluid state to a semicrystalline phase. Consequently, under low temperatures, membranes become less fluid and gel-like, leading to water and soluble material leakage into intercellular spaces. Cold tolerance relating to lipid unsaturation has been noted in many plant species (Atayee and Noori, 2020). A higher proportion of unsaturated fatty acids and a lower transition temperature are associated with increased cold tolerance. Cold-tolerant species boost the proportion of unsaturated fatty acids in their plasma and chloroplast membranes, preserving fluidity and preventing membrane rigidification in low temperatures. Additionally, compatible solutes such as proline, glycine betaine, and soluble carbohydrates accumulate in cells, safeguarding proteins and membranes against dehydration caused by low temperatures.

The tolerance of plants to cold stress involves not only alterations in plasma membrane fluidity and osmotic regulators, but also increased antioxidant production and hormonal regulation, which are integrated with cold-induced signalling cascades. Some plant species change their gene expression patterns and alter the functions of stress-related proteins and metabolic pathways through gradual exposure to low temperatures, thereby increasing their cold tolerance in a process called cold acclimation (Fürtauer et al., 2019). Low temperature induces ROS production; thus, effective cold stress tolerance involves increased antioxidant production to mitigate ROS-induced oxidative damage. Similarly, efficient hormonal regulation promotes growth and development at low temperatures. Hormones such as ABA, gibberellins, and cytokinins are key regulators of plant responses to cold stress (Ashraf and Rahman, 2018). While gibberellins and cytokinins are reported to regulate growth and development under low temperature, ABA promotes the expression of cold-responsive genes like OsDREB1A and OsDREB1BI rice, COR15A, COR47, KIN1 and CBF/DREB1 in Arabidopsis (Zhou et al., 2025). Reproductive development is highly susceptible to cold stress, and plants' capacity to withstand low temperatures during this period influences the stability of their grain supply. In rice, the capacity to endure low temperatures during the booting stage is correlated with increased anther size, increased pollen quantity, and improved pollen yield. Changrong et al. (1998) demonstrated that tolerant rice cultivars produced a greater quantity and size of viable pollen grains, resulting in enhanced reproductive success. Sanghera and Wani, (2008) reported that rice cultivars capable of withstanding low temperatures have spikelet fertility above 90% and well-exserted panicles under temperate conditions, indicating their ability to maintain substantial yields despite lower temperatures. Tolerance traits have also been documented in many other crop species. In wheat, cold tolerance is correlated with the synthesis of CBF/DREB transcription factors, which regulate downstream COR genes and improve winter survival (Yahia et al., 2018). Cold-tolerant maize genotypes exhibit improved photosystem II efficiency and chlorophyll stability under chilling conditions (Li et al., 2019). Legumes, including faba bean (Vicia faba) and lentil (Lens culinaris), exhibit genotype-specific tolerance traits, such as increased pollen viability and stable seed development during early-season frosts (Bhat et al., 2022). Cold tolerance comprises structural integrity, metabolic adaptability, and gene regulatory networks that enable plants to sustain both vegetative and reproductive functions despite experiencing cold and freezing stress.

## 3 Biochemical basis of cold stress tolerance in plants

Plants have developed intricate biochemical strategies to withstand low temperatures, helping them preserve cellular integrity and physiological functions under stress (**Figure 1**). These biochemical changes are vital for protecting cells, maintaining enzyme activity, and stabilising metabolism in cold conditions (Adhikari et al., 2022). During cold stress, plants produce various cryoprotective components, including soluble sugars such as glucose, fructose, sucrose, and galactose; amino acids like alanine, glycine, proline, and polyamines (PAs), notably putrescine and spermidine; and numerous secondary metabolites, including phenols, flavonoids, coumarins, catechins, and tocopherols (Guo et al., 2018). All these cryoprotectants generated at low temperatures help to enhance membrane stability, regulate osmotic potential, scavenge reactive oxygen species (ROS), and prevent ice crystal formation. To survive cold conditions, plants also synthesise various proteins, including antifreezing proteins, late embryogenesis abundant proteins, and cold shock proteins (Kazemi-Shahandashti and Maali-Amiri, 2018). Likewise, low temperatures trigger the production of stress-related hormones and enzymes that initiate cold-responsive signalling cascades to mitigate the harmful effects of stress. Hormones such as abscisic acid, gibberellin, and jasmonic acid, alongside enzymes like protein kinases, collectively regulate plant responses to cold stress.

### 3.1 Lipid and membrane stability

Modifying lipid composition to maintain membrane integrity and fluidity is a vital biochemical process that influences a plant's ability to withstand cold stress. During cold conditions, the fluidity of cellular membranes typically decreases, causing them to become gel-like and semicrystalline. This hampers the movement of ions across membranes and disrupts cellular metabolic activities. To counter this, cold-resistant plants adapt their membrane lipid composition, mainly by increasing the proportion of unsaturated fatty acids (Mazur et al., 2020). However, while unsaturated fatty acids help retain membrane fluidity at lower temperatures, saturated fatty acids tend to solidify quickly. A group of enzymes, especially fatty acid desaturases (FADs), drives this remodelling process. They introduce double bonds into fatty acid chains, lowering the membrane’s phase transition temperature. Proteins such as lipid transfer proteins (LTPs), phospholipases, and desaturase-related regulators support changes in membrane structure during cold and freezing stress. This ensures the continued operation of transport systems, signalling pathways, and protein‒lipid interactions (Wani et al., 2018). Besides their structural role, membrane lipids act as precursors for lipid-derived signalling molecules such as phosphatidic acid (PhAc), inositol phosphates, and oxylipins. These molecules function as second messengers in pathways that respond to cold stimuli. They activate genes that facilitate cold adaptation, modify antioxidant system activities, and interact with phytohormonal signalling networks, especially those involving abscisic acid (ABA) and jasmonic acid (JA). The lipid-based signalling cascade is dynamic, showing that it can change over time (Shomo et al., 2024). It supports cold acclimation, the gradual increase in tolerance to low but nonfreezing temperatures, and deacclimation, the reversal to growth-favouring conditions when temperatures rise. Lipid and membrane stability are essential metabolic processes that help plants adapt to cold stress by strengthening their structure and triggering complex signalling pathways.

### 3.2 Osmoprotectants

Osmoprotectants are vital for the metabolic basis of cold stress tolerance because they protect plant cells from osmotic imbalance and dehydration, which can occur as temperatures drop. Cold stress often causes cells to absorb less water and form ice externally, leading to water loss, reduced turgor, and destabilisation of macromolecules. Plants produce various suitable solutes, such as sugars (sucrose, trehalose, and raffinose family oligosaccharides), amino acids (proline), and quaternary ammonium compounds (glycine betaine), to counteract these effects (Govrin et al., 2019). These solutes not only affect cellular metabolism but also aid in osmotic regulation and cryoprotection. They help maintain protein and membrane stability, prevent protein aggregation, and inhibit enzyme degradation at low temperatures (Pattnaik et al., 2021). Raffinose family oligosaccharides are known for preserving membrane flexibility and protecting thylakoid membranes in chloroplasts from freezing stress (Gu et al., 2018). Sucrose interacts with lipid headgroups, directly stabilising plasma membranes during cold stress (Wingler et al., 2020). Likewise, fructose-based oligosaccharides and polysaccharides are incorporated between the polar headgroups of plant cell membranes stressed by cold. The detection of fructans in the apoplast at low temperatures indicates their role in vesicle-mediated transport (Tarkowski and Van den Ende, 2015). Therefore, the subcellular levels of soluble sugars fluctuate in response to optimal temperatures in plants.

Proline, a potent biomarker for plant cold tolerance, plays vital roles in stabilising cell membranes, scavenging ROS-induced oxidative stress, and regulating osmotic potential. It also functions as both an osmolyte and a molecular chaperone, preventing improper protein folding (Raza et al., 2023). Glycine betaine, another well-known stress protectant, helps regulate redox balance and osmotic potential (Zulfiqar et al., 2022). Cold-responsive genes and transcription factors involved in proline and glycine betaine biosynthesis meticulously oversee the production and storage of osmoprotectants (Dikilitas et al., 2020). These genes and factors are activated as the plant acclimates to cold temperatures. Hormonal signalling, especially that of ABA, further influences the activation of osmoprotectant production pathways (Lim and Lee, 2020). Likewise, polyamines such as putrescine, spermidine, and spermine actively participate in plant cold stress tolerance mechanisms (Upadhyay et al., 2020). Under low temperatures, PAs help stabilise the photosynthetic apparatus, maintain redox homeostasis, improve membrane stability, and promote osmolyte accumulation (Gupta et al., 2013). Besides their structural and osmotic roles, several osmoprotectants contribute to antioxidant defences by neutralising the ROS that accumulate during chilling or freezing. Osmoprotectants aid plants in enduring cold conditions by protecting cells from desiccation, maintaining membrane stability, regulating redox processes, and supporting metabolic balance. This makes them essential metabolic buffers that help plants adapt to and withstand prolonged exposure to low temperatures.

### 3.3 Oxidative stress and the antioxidative defense system

Cold stress significantly impairs plant metabolism by disrupting cellular homeostasis and damaging electron transport chains in chloroplasts and mitochondria. This leads to excessive production of reactive oxygen species (ROS), including superoxide radicals (O₂⁻•), hydrogen peroxide (H₂O₂), hydroxyl radicals (•OH), and singlet oxygen (¹O₂), in plants (Dreyer and Dietz, 2018). At low concentrations, ROS function as signalling molecules that help plants adapt to stress. However, when they accumulate excessively during cold conditions, they cause oxidative stress, damaging lipids, proteins, nucleic acids, and cells overall. Plants have evolved a sophisticated antioxidant system, comprising both enzymatic and non-enzymatic components, to defend against such damage (Gupta et al., 2018). Superoxide dismutase (SOD) is a vital antioxidant enzyme that converts superoxide into hydrogen peroxide. Catalase (CAT) and ascorbate peroxidase (APX) then decompose H₂O₂ into water. Glutathione reductase (GR) maintains cellular redox balance through the ascorbate–glutathione cycle (Fujita and Hasanuzzaman, 2022). Cold-acclimated Arabidopsis thaliana displays increased levels of Cu/Zn-SOD, APX1, and CAT1, while heightened APX and GR activity are linked to freezing tolerance in both wheat and barley (Filiz et al., 2019). The synergistic action of ROS-scavenging enzymes and metabolites not only prevents cellular damage from oxidative stress but also maintains ROS signalling balance. This allows plants to detect cold stress, initiate metabolic adjustments, and activate defensive responses. Simultaneously, nonenzymatic antioxidants such as ascorbic acid, glutathione, carotenoids, flavonoids, and tocopherols act as effective ROS scavengers, protecting membranes, proteins, and DNA from oxidative damage, while stabilising photosynthetic pigments (Abdulfatah, 2022). Cold stress induces the transcription of numerous antioxidant defence genes. Many of these genes are regulated by cold-responsive transcription factors, including the CBF/DREB, NAC, and WRKY families, ensuring rapid and adaptive responses to oxidative imbalances (Hwarari et al., 2022). The antioxidative defence system is essential for plant survival during cold stress, serving as a critical biochemical barrier that mitigates oxidative damage and helps plants sustain growth and reproductive success in low-temperature environments.

### 3.4 Phytohormones

Plant hormones play essential roles in controlling how plants respond to cold stress at both biochemical and molecular levels. They serve as the main regulators of stress detection, signal transduction, and adaptive responses, which involve activating the antioxidant enzyme system and stress response pathways. Phytohormones such as abscisic acid, ethylene, jasmonic acid, salicylic acid, and gibberellic acid are involved in cold stress signalling and regulating the transcription of genes related to cold stress (Eremina et al., 2016). While the biosynthesis of abscisic acid and jasmonic acid increases under low temperature, the levels of ethylene and gibberellic acid decrease during cold stress. Moreover, phytohormones aid in ROS scavenging and the metabolism of osmoprotectants.

#### 3.4.1 Abscisic acid

ABA is a key compound that helps plants cope with cold stress. ABA acts as a main regulator, connecting environmental cold signals with metabolic processes that protect the organism. It boosts the production of osmoprotectants such as proline and soluble carbohydrates, which aid in osmotic regulation and cryoprotection, thus providing defence against dehydration and oxidative harm caused by low temperatures (Huang et al., 2017). ABA also facilitates stomatal closure, reducing water loss during cold-induced dehydration, and increases the activity of antioxidant enzymes to defend against oxidative stress from cold exposure. The cold-triggered build-up of ABA promotes the expression of several COR genes, many of which are controlled by the ABA-dependent C-repeat binding factor/dehydration-responsive element-binding (CBF/DREB) pathway (Rubio et al., 2019). Furthermore, ABA encourages the synthesis of late embryogenesis abundant (LEA) proteins and dehydrins, which help stabilise membranes and proteins under cold conditions.

#### 3.4.2 Ethylene

Ethylene, a stress-responsive hormone, has dual roles in helping plants cope with cold stress. Ethylene can either increase or decrease plant tolerance, depending on the species and their stress levels (Sharma et al., 2019). Cold stress often triggers ethylene production, which then regulates cold-induced gene expression [e.g., CBF1 (DREB1b), CBF2 (DREB1c), CBF3 (DREB1a)] and alters membrane fluidity. In Arabidopsis, ethylene signalling enhances cold resistance by regulating CBF expression; however, in some cereals, excessive ethylene may inhibit growth. Ethylene influences signalling pathways for ROS, aiding plant responses to stress and balancing growth and defence (Chen et al., 2021).

#### 3.4.3 Jasmonic acid

JA plays a vital role in controlling how plants respond to cold stress, mainly by adjusting the levels of secondary metabolites and protective proteins (Raza et al., 2021). JA signalling triggers cold-responsive transcription factors and stress-inducible genes like OsMYB4, OsAP37, SlMYB15, SlLOXD, SlMYC2, COR15A, COR47, KIN1, etc. It boosts antioxidant defences and lipid remodelling. The build-up of jasmonic acid during cold stress is linked to higher amounts of unsaturated fatty acids in membranes, which enhances fluidity and stability at low temperatures. JA works effectively with ABA; however, it can also inhibit ethylene signalling (Wang et al., 2020; Roychowdhury et al., 2024a). These findings highlight the complexity of phytohormonal interactions that influence plant cold tolerance.

#### 3.4.4 Salicylic acid

Salicylic acid (SA), a naturally occurring phenolic compound, plays a crucial role in the response to abiotic stresses, including cold stress (Mishra et al., 2024; Roychowdhury et al., 2024b). In Arabidopsis, both endogenous free salicylic acid and glucosyl salicylic acid levels increase in response to low temperatures (Wu et al., 2019). Similar findings in wheat and grape berries suggest a potential role for salicylic acid in plant cold tolerance (Aazami et al., 2014). Additionally, the external application of SA has been reported to alleviate chilling stress in winter wheat and green bell pepper, and to enhance cold stress tolerance in rice, maize, and cucumber (Xu et al., 2025). SA is vital in regulating various cold stress-induced signalling pathways, such as ABA-dependent and ABA-independent pathways, Ca2+ signalling, MAPK pathways, and reactive oxygen species (ROS) pathways (Mishra et al., 2024; Roychowdhury et al., 2024b). Moreover, salicylic acid has been shown to activate antioxidant systems and heat shock proteins in peach (*Prunus persica* L.) to reduce chilling injury (Zhang et al., 2024b).

#### 3.4.5 Gibberellic acid

Gibberellic acid is an essential plant hormone typically linked to promoting growth, seed germination, and flowering (Shah et al., 2023). Its levels often decline when temperatures drop. Low temperatures reduce GA production and signalling, thereby limiting excessive growth and conserving energy for survival. DELLA proteins, which suppress GA signalling, become activated by cold stress. This activation inhibits cellular proliferation and slows reproductive growth (Shahan, 2020). DELLA-interacting growth regulatory factors modulate plant growth under cold stress. However, GA is crucial for recovery from cold stress, as it aids in resuming development when temperatures rise. In some instances, controlled GA signalling enhances cold tolerance by increasing the expression of cold-responsive genes (such as CBF1/2/3, COR15A/47/KIN1, WRKY53, ABI5, etc.) alongside ABA and auxin (Colebrook et al., 2014). GA acts as a negative regulator of growth during cold stress and as a positive regulator following deacclimation.

#### 3.4.6 Auxin (IAA)

It is essential for regulating plant growth and developmental plasticity in response to decreasing temperatures, but its role is more complex than that of ABA. Low temperatures can disrupt auxin transport and signalling, altering root morphology and inhibiting cellular growth (Bielach et al., 2017). For instance, reducing the activity of PIN-FORMED (PIN) auxin efflux transporters impairs auxin mobility in root tips, thereby slowing growth under cold conditions. Auxin promotes cold tolerance in plants by modifying cellular mechanisms involved in wall reconstruction and the initiation of lateral roots (Tiwari et al., 2023). This supports nitrogen absorption in plants during stress periods. The interaction between auxin and ABA is vital, as ABA often counteracts auxin-mediated development to prioritise survival over growth. Auxin signalling under cold stress demonstrates a carefully regulated interplay between growth inhibition and cellular function modification for adaptation.

### 3.5 Hormonal interactions and cross-regulation during plant cold acclimation

The ability of plants to manage cold stress is regulated by a highly dynamic and interconnected hormonal network. Auxin, abscisic acid, gibberellic acid, jasmonic acid, and ethylene interact both synergistically and antagonistically to maintain a balance between growth and stress adaptation (Aslam et al., 2022). As temperatures decrease, ABA levels rapidly rise, triggering cold-responsive signalling cascades that lead to the accumulation of osmoprotectants, enhanced antioxidant defences, and activation of CBF/DREB transcription factors. In this context, ABA often opposes auxin by inhibiting its transport and distribution through the regulation of PIN proteins. This limits cellular elongation and developmental expansion to prioritise stress survival. ABA significantly inhibits GA synthesis and stabilises DELLA proteins. This halts developmental processes such as germination and flowering, allowing energy to be redirected towards stress defences (Jahed et al., 2023). Conversely, ABA and JA generally cooperate as both hormones activate genes that respond to cold, increase membrane stability, assist cells in adapting to osmotic changes, and eliminate ROS. This enhances cellular resilience against damage caused by freezing (Yu et al., 2020). However, ethylene plays a more complex role. It can collaborate with ABA and JA to produce stress-responsive transcription factors; nonetheless, excessive ethylene can accelerate ageing and intensify oxidative damage, requiring careful regulation of its levels (Shi and Yang, 2014). Ethylene interacts with auxin to modify root morphology and flexibility during cold stress. Its interaction with GA may also inhibit growth during prolonged chilling. These cross-regulatory interactions work together to create a finely tuned hormonal network (Kazan, 2015). ABA acts as the main regulator, modulating growth-promoting hormones such as auxin and GA, and collaborating with JA and occasionally ethylene to enhance stress tolerance (Golldack et al., 2013). This intricate hormonal signalling framework helps plants cope with immediate cold stress effects and resume normal growth and development once conditions improve (Barrero-Gil and Salinas, 2017). Such mechanisms are vital in determining crop adaptability to cold conditions and their resilience in such environments.

### 3.6 Use of exogenous protectants in mitigating cold-induced damage

The use of exogenous protectants has proven effective in safeguarding plants against cold-induced damage by increasing their physiological and biochemical resilience to low temperatures. Numerous chemicals, including osmoprotectants, antioxidants, plant hormones, and signalling molecules, can be applied externally to enhance the innate resilience systems of plants in response to cold and freezing conditions (Feng et al., 2025b). Osmolytes such as proline, glycine betaine, and soluble carbohydrates, when added outside cells, facilitate osmotic balance, stabilise proteins and membranes, and prevent cellular desiccation under cold stress (Zulfiqar et al., 2020). Antioxidants, including ascorbic acid, glutathione, and tocopherols, are essential because they eliminate excess ROS, thereby protecting chloroplasts and mitochondria from significant oxidative damage. When applied topically, plant hormones can modify plant responses to cold stress (Cai et al., 2018). ABA enhances stomatal regulation and osmoprotectant production, while SA and JA activate genes that support plant defence and improve photosynthetic efficiency under cold conditions (Kosakivska et al., 2022). Signalling molecules such as nitric oxide (NO), hydrogen sulfide (H₂S), and polyamines protect cells by maintaining redox balance, activating stress-responsive transcription factors, and stabilising membranes (Pirzadah et al., 2013). Recent research has highlighted the effectiveness of exogenous melatonin and brassinosteroids in boosting antioxidant activity, maintaining chloroplast integrity, and fostering overall cold tolerance (Fu et al., 2022). Exogenous melatonin can shield plants from cold-induced reactive oxygen species (ROS)-mediated lipid peroxidation, which is increased by abscisic acid (ABA) through enhanced antioxidant activity. The method of applying these protectants can include soaking via roots, foliar spraying, or seed priming, which significantly impacts their effectiveness. The simultaneous application of exogenous protectants is a viable, cost-effective, and environmentally sustainable approach to increasing crop resistance to cold stress (Elkelish et al., 2020). This safeguards yield and quality in regions vulnerable to low temperatures. Such approaches are especially beneficial for climate-smart agriculture, where improving crop resilience to abiotic stresses is vital for a sustainable food supply.

## 4 Signal transduction and gene expression in plants under cold stress

**Figure 3:**
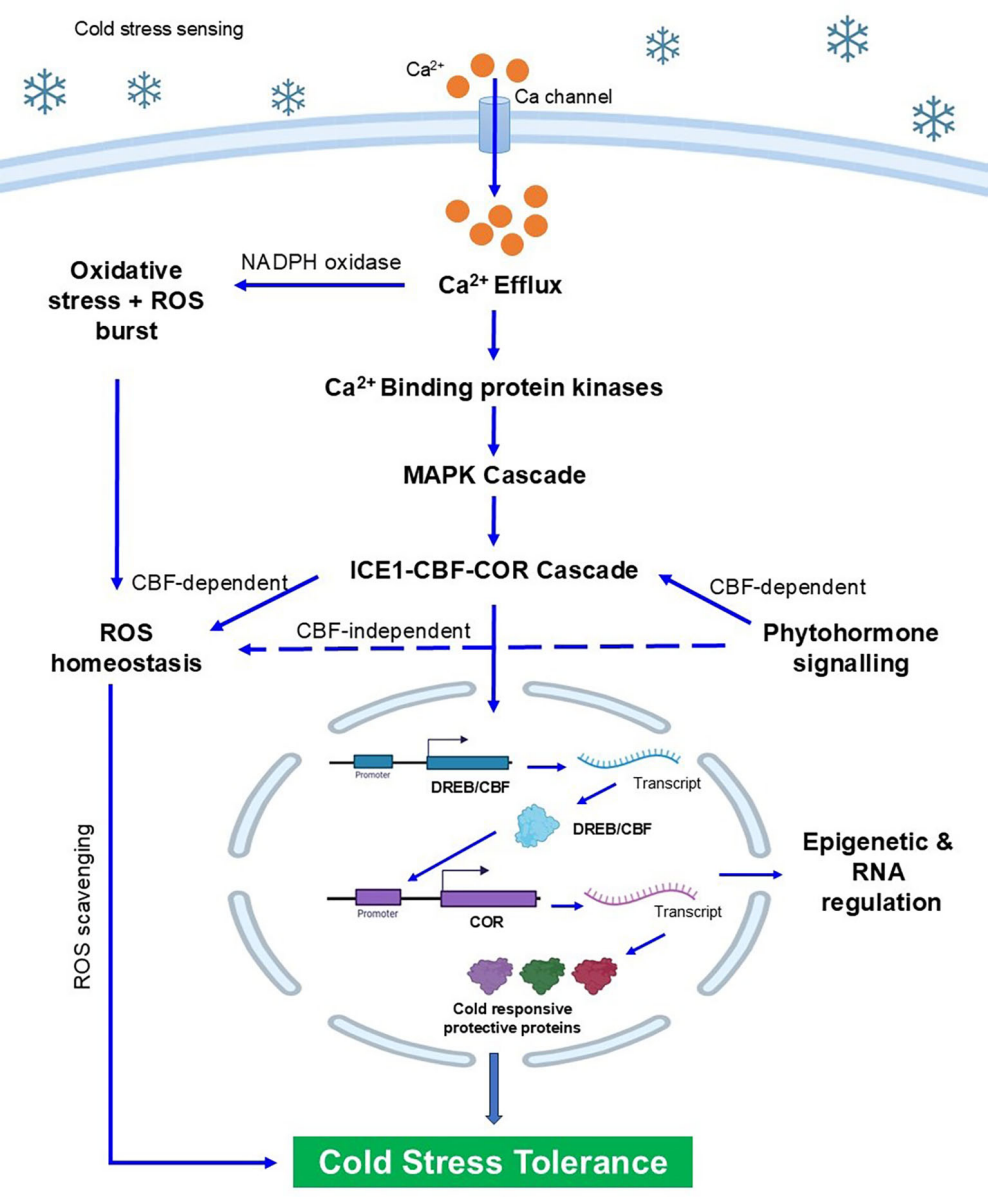
Diagram depicting strategies for adaptation (A) and tolerance (T) to cold stress, organized in a tree diagram. Nodes labeled A01 to A04 include morphological changes, modulation in conductivity, delayed phenology, and alteration of membrane lipid composition denotes adaptive strategies. Nodes labeled T05 to T08 include osmoprotectants, antioxidant defense, signal transduction and transcriptional regulation, and expression of stress-responsive proteins denotes tolerance mechanism

Cold stress in plants is detected through a series of overlapping biophysical, biochemical, and molecular events, happening almost immediately after exposure to low temperatures. The initial detection occurs at the plasma membrane, where a decrease in membrane fluidity serves as the first biophysical cue. This change triggers the opening of calcium-permeable channels, mechanosensitive proteins, and receptor-like kinases (RLKs), which are the primary transducers of cold signals. The rapid rise in cytosolic Ca²⁺ levels acts as a second messenger, conveying the signal’s strength, timing, and pattern (Sardoei and Fazeli-Nasab, 2025). The calcium signals produced in response to cold stress are recognised by calcium-binding proteins such as calmodulins (CaMs), calcineurin B-like proteins (CBLs), and calcium-dependent protein kinases (CDPKs), which then phosphorylate downstream proteins, initiating a response to cold stress (**Figure 3**).

Alongside these effects, cold stress induces a rise in the production of reactive oxygen species (mainly in chloroplasts and the apoplast), which are harmful entities and secondary messengers. In addition to calcium waves, ROS generation activates phosphorylation pathways, especially the mitogen- activated protein kinase (MAPK) module. The MEKK 1–MKK 2–MPK 4/6 cascade has been identified as a key component in cold signalling, regulating transcription factors' activity and coordinating the expression of COR genes (Zhou et al., 2025). A list of various potential cold- responsive genes is shown in **Table 1** with their respective crop species. Crosstalk between ROS, NO, and lipid- derived signals helps amplify transcriptional reprogramming, which is essential for acclimation. Hormonal signalling adds an extra layer of regulation. ABA responds through activation of the ABA- responsive element binding factor (ABF/AREB) and the induction of COR gene expression. JA and SA similarly act in concert or opposition to ABA within transcriptional cascades. For example, cold- triggered ICE 1 stabilization by JA increases CBF levels, and SA can fine- tune ROS homeostasis under cold conditions (Das et al., 2025). Ethylene signalling, mediated by EIN 3/EIL 1, generally represses CBF expression, demonstrating the complex hormonal cross- regulation that tightly manages the cold response. At the transcriptional level, the AP 2/ERF superfamily, especially the C- Repeat binding factor (CBF)/dehydration- responsive element binding protein (DREB) superfamily, forms the central regulatory hub for cold acclimation. Once activated by ICE 1 and ICE 2, these factors bind to a CRT/DRE (A/GCCGAC) cis- element in the promoter region of COR genes (including RD 29 A, COR 15 A, COR 47, and KIN 1) (Nakashima et al., 2025). The expression of COR genes stabilises membranes, promotes osmolyte accumulation (such as proline, raffinose, and sugars), and enhances antioxidative capacity, collectively increasing cold tolerance. Notably, ICE 1 undergoes posttranslational modifications through phosphorylation (by OST 1/Snrk 2 kinases), ubiquitination (by the HOS 1 E 3 ligase), and SUMOylation (by SIZ 1), which dynamically regulate the levels of CBF expression (Zhang et al., 2025).

**Table 1:** Summary of key cold-regulated (COR) genes, transcription factors (TFs), and signaling components involved in cold stress response across major crop species.

Transcription Factors (TFs)	Signaling Components	References
CBF1, CBF2, CBF3	ICE1, MAPK cascade	Thomashow (1999), Jeon and Kim (2013)
OsCBF1, OsNAC6	OsICE1, OsMAPK5	Dubouzet et al. (2003); Nakashima et al. (2009)
ZmCBF3, ZmMYB	ZmMPK3, ZmICE1	Qin et al. (2004), Zhou et al. (2022)
TaCBF1, TaNAC2	TaMAPK, TaICE1	Soltani et al. (2022), Yu and Zhang (2023)
GmDREB1, GmMYB76	GmMAPK3, GmICE1	Kidokoro et al. (2015), Liao et al. (2008)
SlCBF1, SlNAC1	SlMAPK, SlICE1	Li et al. (2025b), Jiao and Sun (2025)

Variation in CBF gene copy number, promoter structure, and expression dynamics contributes to the diversity in cold tolerance among species and accessions (Li et al., 2025a). For example, tolerant accessions of Brassica rapa show late but prolonged CBF induction, whereas the expression of Oscbf 1–3 varied greatly between Indica (93--11) and Japonica (Nipponbare) rice cultivars because of cis- regulatory polymorphisms (Sun et al., 2020). These differences largely demonstrate how evolutionary divergence at the transcriptional regulation level has shaped species- specific cold acclimation responses. Besides CBFs, numerous other transcription factor families participate in cold signalling, creating significant redundancy and complementarity within the network. WRKY proteins are involved in an ABA- dependent pathway by binding W- box cis elements and regulating genes like ABI 5 and COR 47. For instance, knockdown of Cswrky 46 reduces chilling sensitivity, while simultaneously promoting proline synthesis and alleviating cellular damage. Similarly, NAC and MYB transcription factors govern lignin deposition, osmoprotectant biosynthesis, and ROS detoxification, adding complexity to the transcriptional control of cold tolerance (Qian et al., 2024). Epigenetic regulation also plays a role in cold tolerance. The accessibility of CBF and COR regulators is controlled by histone modifications (e. g., H 3 K 4 me 3 activation and H 3 K 27 me 3 repression) and chromatin remodellers (Hereme et al., 2021). Furthermore, DNA methylation, in conjunction with cold response long non- coding RNAs and microRNAs (such as mir 169, mir 319, and mir 393), contribute to adjusting transcript stability and translation efficiency. These epigenetic and RNA- based mechanisms collectively enable stress memory in plants, facilitating rapid and substantial responses upon cold recurrence (Vaschetto, 2024). Three categories of functional proteins are involved in cold stress adaptation: (a) signal sensors and transducers—Ca ²⁺ sensors, CDPKs, MAPKs, and RLKs; (b) transcription factors—ICE 1/2, CBFs, WRKYs, NACs, MYBs, and ABFs; and (c) protective effectors—LEA proteins, dehydrins, molecular chaperones, enzymes involved in osmolyte biosynthesis, and antioxidants (SOD, APX, and CAT). Overall, these multi- layered regulatory networks indicate that cold stress perception is not merely a linear cascade but a complex, dynamic, and extensively interconnected system that integrates signal perception with transcriptional reprogramming, epigenetic regulation, and hormonal cross- talk to optimise plant survival under fluctuating low temperatures.

## 5 Metabolic reprogramming and stress memory in plants for cold stress tolerance

Cold stress is a major factor that threatens plant survival and productivity by disrupting cellular homeostasis and metabolism. When exposed to cold, plants globally reprogram their metabolism so that different types, including primary and secondary, are dynamically adjusted, and energy and carbon metabolism are selectively readjusted (Fürtauer et al., 2019). These changes are essential to maintain cellular structure, support growth, and trigger defensive mechanisms in response to cold stress. The key metabolic pathways involved in plant growth and development are mostly affected by cold stress (Xu and Fu, 2022). One of the earliest responses is changes in carbohydrate metabolism, especially in the levels of soluble sugars like sucrose, glucose, and raffinose. These sugars serve multiple functions, such as osmoprotectants, membrane stabilisers, and signalling molecules that activate stress-responsive genes. Additionally, cold stress causes alterations in amino acid profiles, with increases in proline, glycine, and alanine. Proline, in particular, plays an important role in osmotic regulation, ROS scavenging, and stabilising proteins (Jahed et al., 2023). Moreover, secondary metabolite profiles are significantly reprogrammed at low temperatures. Phytoprotectants, which promote the biosynthesis of phenolic compounds, flavonoids, and alkaloids, are vital in boosting antioxidant defences and stabilising membranes in plants. These metabolites are not only used to counter oxidative damage but also function as signals within stress-response cascades that help regulate the reaction to stress. For instance, anthocyanins produced in response to cold stress protect photosystems and help maintain redox balance (Rao and Zheng, 2025).

Cold stress is a metabolic limitation caused by a decrease in enzymatic reaction rates and photosynthetic activity. To acclimate, plants divert carbon from growth to mitigate stress. This process involves shutting down high-energy biosynthetic activities and activating catabolic processes, which produce ATP and reduce the equivalents needed for stress responses. Typically, mitochondrial respiration is adjusted for energy needs, and the activity of alternative oxidase (AOX) pathways is sometimes increased to lower ROS (Monteiro-Batista et al., 2025). Under cold stress, chloroplast metabolism shifts from its primary role in carbon fixation to producing protective and defensive compounds, aiding plant adaptation to challenging conditions. Additionally, the expression of starch catabolism genes rises to facilitate their breakdown for cellular defence. Lipid metabolism is also reorganised to preserve membrane fluidity, with plants adjusting the unsaturation levels of membrane lipids to maintain functionality at low temperatures. This lipid reorganization is vital for organelle maintenance and signal transduction during cold stress (Bhattacharya, 2022b).

Specialized metabolites that directly participate in stress adaptation are synthesised by plants in response to cold stress. These osmoprotectants (i.e., polyamines, glycine betaine, and trehalose) stabilise membranes and proteins. Polyamines such as putrescine and spermidine help control ion channels, antioxidative enzymes, and gene expression during chilling. Their accumulation in chloroplasts also aids in maintaining the efficiency of photosynthesis by protecting the photosystem II (PSII) complex (Wei et al., 2022). Trehalose is a nonreducing disaccharide, and it acts as a signalling molecule and protectant that safeguards protein conformation and prevents aggregation against freezing stress. Furthermore, cold stress induces the production of VOCs and terpenoids, which may be involved in intercellular communication and priming defence. These secondary metabolites not only enable the plant to be frost-hardy but also prepare the plant for other future stress events, a concept they refer to as ‘metabolic memory’ (Gusain et al., 2025).

Metabolic memory describes the phenomenon whereby plants retain a physiological and biochemical response to environmental stresses, such as cold, even after the stressor has been removed. This capacity to "remember" previous stress experiences enables plants to respond more quickly and effectively to subsequent stress events, enhancing their overall resilience (Maric, 2025). In the context of cold stress, metabolic memory is vital in preparing plants for potential future encounters with low temperatures, ensuring they can promptly activate protective mechanisms. The formation of metabolic memory is primarily associated with changes in gene expression and epigenetic modifications (Roychowdhury et al., 2023a). After an initial cold stress event, specific genes related to cold tolerance may be upregulated or downregulated, leading to a lasting change in the plant's physiological state. These alterations can persist over time, allowing plants to respond more rapidly to future cold exposure. Additionally, epigenetic modifications, such as DNA methylation and histone modifications, further support the sustained expression of stress-responsive genes. This molecular memory allows plants to stay in a heightened state of readiness, improving their ability to withstand subsequent cold stress. Stress memory, closely related to metabolic memory, encompasses the broader capacity of plants to retain knowledge of past stress conditions and adjust their metabolic responses accordingly (Ramakrishnan et al., 2022). This ability is especially important in cold stress tolerance. Stress memory mechanisms are mediated by complex signalling networks involving ABA, JA, and SA, as well as secondary metabolites. These signalling molecules play crucial roles in reinforcing physiological changes initiated during the initial stress, enabling plants to mount a more effective defence against future cold events. During cold stress, metabolic reprogramming takes place as plants undergo significant shifts in metabolic pathways to maintain cellular homeostasis and ensure survival. One key change involves carbohydrate metabolism, where an increase in soluble sugars such as sucrose and glucose acts as an osmoprotectant (Roychowdhury et al., 2025a). These sugars help stabilise cellular structures and provide energy during recovery from cold stress. Furthermore, starch degradation is enhanced, supporting energy needs amid low temperatures. Lipid metabolism also undergoes significant modifications during cold stress. Changes in membrane lipid composition, including the accumulation of unsaturated fatty acids, increase membrane fluidity, which is essential for maintaining cellular integrity under low-temperature conditions. Moreover, stress-induced lipid signalling molecules can activate cold-responsive genes, aiding plant adaptation to chilling stress. Amino acid and protein metabolism are also notably affected. The synthesis of certain amino acids, such as proline and glycine betaine, rises during cold stress, contributing to osmotic adjustment and safeguarding cellular structures from damage. Additionally, the expression of heat shock proteins (HSPs) and other chaperones is stimulated, assisting in protein folding and repair mechanisms vital for cellular function during and after cold exposure (Story and Storey, 2023). Antioxidant defence mechanisms also play a crucial part in metabolic reprogramming during cold stress. The production of ROS often increases under cold conditions, necessitating the upregulation of antioxidant enzymes, including superoxide dismutase, catalase, and peroxidase. These enzymes reduce oxidative damage and help to maintain cellular redox balance. Metabolites such as ascorbate and glutathione are also essential for the proper functioning of these antioxidant systems (Jahed et al., 2023). The interaction between metabolic memory and stress memory provides plants with a strong strategy for coping with cold stress. Understanding these processes offers valuable insights into the underlying mechanisms of plant resilience and paves the way for developing strategies to enhance cold tolerance in crops. Future research should aim to elucidate the molecular mechanisms underpinning these memory processes, identify key genes and pathways involved, and employ biotechnological tools to manipulate metabolic pathways for improved cold stress tolerance.

## 6 Translating cold stress tolerance in breeding and crop improvement

Although significant progress has been made in understanding the physiological, biochemical, and molecular basis of cold tolerance, applying this knowledge to breeding programmes remains a major challenge for developing climate-resilient crops. Identifying traits that enhance resilience at subzero temperatures or under cold stress is the first step towards a targeted breeding approach. These traits may include membrane fluidity, cellular antioxidant capacity, osmoprotectant synthesis, hormonal homeostasis, and the efficiency of photosynthesis under cold-stressed conditions. Phenotypic and morphological traits such as spikelet or floral fertility (in field crops), pollen viability, chlorophyll retention, and seedling vigour under chilling or freezing conditions are effective and reliable indicators of tolerance (Ren et al., 2022). High-throughput phenotyping platforms, such as imaging and chlorophyll fluorescence-based assays, facilitate precise measurement of these traits across different crop species. The combined use of physiological and biochemical indices with phenotypic data supports the selection of cold-tolerant lines. Furthermore, in terms of genetic resources and germplasm utilisation, wild relatives and landraces act as reservoirs of cold tolerance traits (Thakur et al., 2024). For example, temperate cereals, including barley and rye, and legumes like faba bean and lentil, naturally adapt to low temperatures. These genotypes often carry specific alleles of cold-responsive genes, such as CBF/DREB TFs, LEA proteins, and antioxidant enzymes. Pre-breeding efforts involving extensive introgression of these traits into elite cultivars are vital for expanding the genetic basis of cold tolerance (Sukumaran et al., 2022). Genebanks and international crop improvement initiatives should focus on characterising and exploiting cold-tolerant germplasms through shared screening and testing efforts. Additionally, advances in molecular genetics have made it easier to identify QTLs and SNPs linked to cold stress tolerance. Marker-assisted selection (MAS) can help breeders track these loci during hybridisation and selection, aiding the development of tolerant varieties (Roychowdhury, 2014). Genomic selection (GS), which utilises genome-wide markers to predict breeding values, is a powerful tool for polygenic traits involved in G×E interactions, such as cold tolerance. In practice, to breed cold-tolerant cultivars, pearl millet is being developed through a concerted effort combining conventional and molecular techniques. Given farmers’ involvement in the production process, partially participatory breeding is essential in cold-prone agro-ecologies to enhance the relevance and acceptance of new varieties. Trait introgression via hybrid breeding, recurrent selection, and backcrossing with tolerant donors has proven successful. Both MAS and GS are molecular breeding approaches that improve selection accuracy and reduce breeding cycles. Integrating omics data into the breeding pipeline enables the identification of high-potential genotypes with increased tolerance and sustainable high yields in cold environments (Roychowdhury et al., 2023b). For effective translational research and field application, laboratory findings must be validated against field conditions to ensure their practical utility for farmers. Cold risk agro-ecological sites are crucial for assessing genotype performance, stability, and adaptation. Genetic improvements are complemented by agronomic practices such as osmoprotectant-primed seeds, antioxidant foliar applications, and sowing date optimisation (Moradi and Siosemardeh, 2023). Decision-support systems and climate models are valuable tools for guiding the adoption of cold-tolerant varieties in regions experiencing increasing temperature variability due to climate change. Ultimately, supportive policies and capacity-building initiatives are essential to transfer cold research from laboratories to farms. Investment in research infrastructure for phenotyping and genotyping, alongside training breeders and researchers in these advanced tools, will accelerate progress. International collaborations and public‒private partnerships can play a vital role in supporting technology transfer and seed variety development (Smyth et al., 2021). Incorporating cold tolerance traits into national breeding priorities and varietal release regulations will promote long-term sustainability and food security.

## 7 Conclusion and future perspective

Cold acclimation is a complex and adaptable protective process that helps plants survive and adjust to low-temperature stress (Roychowdhury, 2014). It is essential for sustaining growth, development, and productivity, especially in temperate and high-altitude agroecosystems where cold stress frequently occurs (Roychowdhury et al., 2020). This review emphasises the intricate interactions between physiological adaptations, biochemical defences, molecular signalling pathways, and metabolic reprogramming, which together support cold stress tolerance in plants.

Key physiological responses, such as stomatal regulation, membrane stabilisation, and osmotic adjustment, are closely linked to biochemical changes, including the accumulation of osmoprotectants, antioxidants, and stress-responsive proteins (Roychowdhury et al., 2025a). These responses are coordinated by complex molecular signalling cascades involving calcium influx, ROS, MAPK pathways, and hormone-dependent regulatory networks (Anumalla et al., 2016). At the core of this regulation is the ICE1–CBF–COR transcriptional module, which activates cold-responsive genes that enhance cellular protection and stress tolerance. Metabolic reprogramming further supports cold resilience by redirecting energy and carbon fluxes towards the synthesis of protective metabolites such as sugars, amino acids, polyamines, and secondary compounds (Roychowdhury et al., 2023a). These metabolic shifts not only reduce cellular damage but also aid long-term acclimatisation and survival during extended cold exposure.

Despite significant advances, several knowledge gaps remain that hinder the full transfer of cold stress biology into crop improvement. Future research should prioritise the discovery of novel regulatory genes and signalling pathways through high-resolution transcriptomics, proteomics, and epigenomics (Roychowdhury et al., 2023a). Understanding species-specific regulatory networks and their evolutionary divergence will assist in designing targeted interventions for cold tolerance. The integration of multiomics approaches—combining genomics, transcriptomics, metabolomics, and phenomics—will enable a systems-level understanding of cold stress responses (Roychowdhury et al., 2023a). This holistic perspective can identify biomarkers and candidate genes that are vital for breeding and functional validation. Furthermore, exploiting the genetic diversity present in wild relatives and landraces offers a promising pathway for identifying unique alleles associated with cold tolerance (Anumalla et al., 2015; Ganie et al., 2016). Prebreeding efforts should aim at introgressing these traits into elite cultivars to broaden the genetic base. Accelerated breeding using molecular tools such as MAS and GS can substantially enhance the efficiency of selecting complex traits like cold tolerance. The development of high-throughput phenotyping platforms will support rapid screening of genotypes under controlled and field conditions. Precision breeding via CRISPR‒Cas-based genome editing provides unprecedented opportunities to modify key regulatory elements—such as promoters and transcription factor-binding sites—to enhance stress resistance without compromising yield (Roychowdhury et al., 2025b). Metabolomics-assisted breeding is another emerging strategy that can guide the selection of tolerant genotypes based on profiling stress-induced metabolites such as proline, glycine betaine, and raffinose. These metabolites not only act as protective agents but also function as signalling molecules within stress response pathways. Using these genes as metabolite-based markers can complement genomic tools within breeding programmes. Translational research must extend beyond laboratory studies to field-level validation across diverse agroclimatic zones. Multiple location trials and participatory breeding approaches involving farmers will ensure the relevance, adaptability, and adoption of cold-tolerant cultivars. Agronomic practices such as seed priming, foliar application of protectants, and optimised sowing schedules can further mitigate cold stress impacts and boost productivity. Finally, policy support and capacity building are crucial to scaling translational outcomes. Strengthening institutional frameworks, investing in cold stress research infrastructure, and training breeders in advanced tools will accelerate progress. The inclusion of cold tolerance traits in national varietal release criteria and breeding priorities will promote climate-resilient agriculture.

## References

[B1] Aazami M. A. Mahna N. Hasani R. N. (2014). Salicylic acid affects antioxidant system of some grape cultivar under cold stress conditions. J. Biodivers. Environ. Sci. 5, 280–290.

[B2] Abdulfatah H. F. (2022). Nonenzymatic antioxidants in stressed plants: A review. J. Univ. Anbar. Pure Sci. 16, 25–37. doi: 10.37652/juaps.2022.176435

[B3] Adam S. Murthy S. D. S. (2013). “ Effect of cold stress on photosynthesis of plants and possible protection mechanisms,” in Approaches to plant stress and their management ( Springer India, New Delhi), 219–226.

[B4] Adhikari L. Baral R. Paudel D. Min D. Makaju S. O. Poudel H. P. . (2022). Cold stress in plants: Strategies to improve cold tolerance in forage species. Plant Stress 4, 100081. doi: 10.1016/j.stress.2022.100081

[B5] Agurla S. Gahir S. Munemasa S. Murata Y. Raghavendra A. S. (2018). “ Mechanism of stomatal closure in plants exposed to drought and cold stress,” in Survival strategies in extreme cold and desiccation: adaptation mechanisms and their applications, (Singapore: Springer Nature), 215–232., PMID: 10.1007/978-981-13-1244-1_12 30288712

[B6] Albertos P. Wagner K. Poppenberger B. (2019). Cold stress signalling in female reproductive tissues. Plant Cell Environ. 42, 846–853. doi: 10.1111/pce.13408, PMID: 30043473

[B8] Anumalla M. Roychowdhury R. Geda C. K. Bharathkumar S. Goutam K. D. Mohandev T. S. S. (2016). Mechanism of stress signal transduction and involvement of stress inducible transcription factors and genes in response to abiotic stresses in plant. Int. J. Recent. Sci. Res. 7, 12754–12771.

[B7] Anumalla M. Roychowdhury R. Geda C. K. Mazid M. Rathoure A. K. (2015). Utilization of plant genetic resources and diversity analysis tools for sustainable crop improvement with special emphasis on rice. Int. J. Adv. Res. 3, 1155–1175.

[B9] Ashraf M. A. Rahman A. (2018). “ Hormonal regulation of cold stress response,” in Cold tolerance in plants: Physiological, molecular and genetic perspectives ( Springer International Publishing), 65–88.

[B10] Aslam M. Fakher B. Ashraf M. A. Cheng Y. Wang B. Qin Y. (2022). Plant low-temperature stress: Signalling and response. Agronomy 12, 702. doi: 10.3390/agronomy12030702

[B11] Atayee A. R. Noori M. S. (2020). Alleviation of cold stress in vegetable crops. J. Sci. Agric. 4, 38–44. doi: 10.25081/jsa.2020.v4.6110

[B12] Baker N. R. (2019). “ Chilling stress and photosynthesis,” in Causes of photooxidative stress and amelioration of defense systems in plants (Boca Raton, USA: CRC Press), 127–154.

[B13] Banerjee A. Roychoudhury A. (2019). “ Cold stress and photosynthesis,” in Photosynthesis, productivity and environmental stress, (UK: John Wiley & Sons Ltd), 27–37.

[B14] Barrero-Gil J. Salinas J. (2017). CBFs at the crossroads of plant hormone signalling in cold stress response. Mol. Plant 10, 542–544. doi: 10.1016/j.molp.2017.03.004, PMID: 28323054

[B15] Bhat K. A. Mahajan R. Pakhtoon M. M. Urwat U. Bashir Z. Shah A. A. . (2022). Low temperature stress tolerance: An insight into the omics approaches for legume crops. Front. Plant Sci. 13, 888710. doi: 10.3389/fpls.2022.888710, PMID: 35720588 PMC9204169

[B16] Bhattacharya A. (2022a). “ Effect of low temperature stress on photosynthesis and allied traits: A review,” in Physiological processes in plants under low temperature stress, (Singapore: Springer Nature), 199–297.

[B17] Bhattacharya A. (2022b). “ Lipid metabolism in plants under low-temperature stress: a review,” in Physiological processes in plants under low temperature stress, 409–516.

[B18] Bielach A. Hrtyan M. Tognetti V. B. (2017). Plants under stress: involvement of auxin and cytokinin. Int. J. Mol. Sci. 18, 1427. doi: 10.3390/ijms18071427, PMID: 28677656 PMC5535918

[B19] Cai T. Meng X. Liu X. Liu T. Wang H. Jia Z. . (2018). Exogenous hormonal application regulates the occurrence of wheat tillers by changing endogenous hormones. Front. Plant Sci. 9, 1886. doi: 10.3389/fpls.2018.01886, PMID: 30622548 PMC6308958

[B20] Cao Y. Hwarari D. Radani Y. Guan Y. Yang L. (2023). Molecular mechanism underlying plant response to cold stress. Phyton 92, 2665–2683. doi: 10.32604/phyton.2023.024929

[B71] Changrong Y. Xinhua L. Dai L. Jianhua Z. (1998). Factors Related to Cold Tolerance at Booting Stage in *Oryza sativa* L. Chinese Journal of Rice Science 12 (1), 6–10.

[B21] Chen H. Bullock J. D.A. Alonso J. M. Stepanova A. N. (2021). To fight or to grow: the balancing role of ethylene in plant abiotic stress responses. Plants 11, 33. doi: 10.3390/plants11010033, PMID: 35009037 PMC8747122

[B22] Cheng J. Li H. Zao H. Huang Z. Hai M. Fan W. (2022). Molecular regulatory mechanism of plant response to cold stress. Field Crop 5, 1–15. doi: 10.5376/fc.2022.05.0001

[B23] Colebrook E. H. Thomas S. G. Phillips A. L. Hedden P. (2014). The role of gibberellin signalling in plant responses to abiotic stress. J. Exp. Biol. 217, 67–75. doi: 10.1242/jeb.089938, PMID: 24353205

[B24] Das S. Shil S. Rime J. Alice A. K. Yumkhaibam T. Mounika V. . (2025). Phytohormonal signalling in plant resilience: Advances and strategies for enhancing abiotic stress tolerance. Plant Growth Regul. 105, 329–360. doi: 10.1007/s10725-025-01279-6

[B25] Dikilitas M. Simsek E. Roychoudhury A. (2020). “ Role of proline and glycine betaine in overcoming abiotic stresses,” in Protective chemical agents in the amelioration of plant abiotic stress: biochemical and molecular perspectives, (UK: John Wiley & Sons Ltd), 1–23.

[B26] Ding Y. Shi Y. Yang S. (2019). Advances and challenges in uncovering cold tolerance regulatory mechanisms in plants. New Phytol. 222, 1690–1704. doi: 10.1111/nph.15696, PMID: 30664232

[B27] Dreyer A. Dietz K. J. (2018). Reactive oxygen species and the redox-regulatory network in cold stress acclimation. Antioxidants 7, 169. doi: 10.3390/antiox7110169, PMID: 30469375 PMC6262571

[B28] Dubouzet J. G. Sakuma Y. Ito Y. Kasuga M. Dubouzet E. G. Miura S. . (2003). OsDREB genes in rice, Oryza sativa L., encode transcription activators that function in drought-, high-salt-and cold-responsive gene expression. Plant J. 33, 751–763. doi: 10.1046/j.1365-313X.2003.01661.x, PMID: 12609047

[B29] Elkelish A. Qari S. H. Mazrou Y. S. Abdelaal K. A. Hafez Y. M. Abu-Elsaoud A. M. . (2020). Exogenous ascorbic acid induced chilling tolerance in tomato plants through modulating metabolism, osmolytes, antioxidants, and transcriptional regulation of catalase and heat shock proteins. Plants 9, 431. doi: 10.3390/plants9040431, PMID: 32244604 PMC7238171

[B30] Elmore R. W. Doupnik J. B. (1995). Corn recovery from early-season frost. J. Produc. Agric. 8, 199–203. doi: 10.2134/jpa1995.0199

[B31] El-Refaee Y. Z. Gharib H. S. Badawy S. A. Elrefaey E. M. El-Okkiah S. A. Okla M. K. . (2024). Mitigating cold stress in rice: a study of genotype performance and sowing time. BMC Plant Biol. 24, 713. doi: 10.1186/s12870-024-05423-8, PMID: 39060959 PMC11282823

[B32] Eremina M. Rozhon W. Poppenberger B. (2016). Hormonal control of cold stress responses in plants. Cell. Mol. Life Sci. 73, 797–810. doi: 10.1007/s00018-015-2089-6, PMID: 26598281 PMC11108489

[B33] Feng Y. Li Z. Kong X. Khan A. Ullah N. Zhang X. (2025a). Plant coping with cold stress: molecular and physiological adaptive mechanisms with future perspectives. Cells 14, 110. doi: 10.3390/cells14020110, PMID: 39851537 PMC11764090

[B34] Feng D. Zhang M. Xu J. Gao Q. Liu J. Li C. . (2025b). Revelation of mechanisms associated with strengthening plant cold tolerance through using exogenous substances. Front. Plant Sci. 16, 1478692. doi: 10.3389/fpls.2025.1478692, PMID: 40260434 PMC12009806

[B35] Filiz E. Ozyigit I. I. Saracoglu I. A. Uras M. E. Sen U. Yalcin B. (2019). Abiotic stress-induced regulation of antioxidant genes in different Arabidopsis ecotypes: microarray data evaluation. Biotechnol. Biotechnol. Equip. 33, 128–143. doi: 10.1080/13102818.2018.1556120

[B36] Fu J. Zhang S. Jiang H. Zhang X. Gao H. Yang P. . (2022). Melatonin-induced cold and drought tolerance is regulated by brassinosteroids and hydrogen peroxide signalling in perennial ryegrass. Environ. Exp. Bot. 196, 104815. doi: 10.1016/j.envexpbot.2022.104815

[B37] Fujita M. Hasanuzzaman M. (2022). Approaches to enhancing antioxidant defense in plants. Antioxidants 11, 925. doi: 10.3390/antiox11050925, PMID: 35624789 PMC9137904

[B38] Fürtauer L. Weiszmann J. Weckwerth W. Nägele T. (2019). Dynamics of plant metabolism during cold acclimation. Int. J. Mol. Sci. 20, 5411. doi: 10.3390/ijms20215411, PMID: 31671650 PMC6862541

[B39] Ganie S. A. Karmakar J. Roychowdhury R. Mondal T. K. Dey N. (2016). An exploratory study on allelic diversity among rice and its wild species as well as relatives with simple sequence repeat and inter simple sequence repeat markers. Indian J. Biotechnol. 15, 357–362.

[B40] George M. F. Burke M. J. (1977). Cold hardiness and deep supercooling in xylem of shagbark hickory. Plant Physiol. 59 (2), 319–325. doi: 10.1104/pp.59.2.319, PMID: 16659841 PMC542389

[B41] Golldack D. Li C. Mohan H. Probst N. (2013). Gibberellins and abscisic acid signal crosstalk: living and developing under unfavourable conditions. Plant Cell Rep. 32, 1007–1016. doi: 10.1007/s00299-013-1409-2, PMID: 23525744

[B42] Govrin R. Obstbaum T. Sivan U. (2019). Common source of cryoprotection and osmoprotection by osmolytes. J. Am. Chem. Soc. 141, 13311–13314. doi: 10.1021/jacs.9b06727, PMID: 31411463

[B43] Gu H. Lu M. Zhang Z. Xu J. Cao W. Miao M. (2018). Metabolic process of raffinose family oligosaccharides during cold stress and recovery in cucumber leaves. J. Plant Physiol. 224, 112–120. doi: 10.1016/j.jplph.2018.03.012, PMID: 29617631

[B44] Guo X. Liu D. Chong K. (2018). Cold signalling in plants: Insights into mechanisms and regulation. J. Integr. Plant Biol. 60, 745–756. doi: 10.1111/jipb.12706, PMID: 30094919

[B46] Gupta K. Dey A. Gupta B. (2013). Plant polyamines in abiotic stress responses. Acta Physiol. Planta 35, 2015–2036. doi: 10.1007/s11738-013-1239-4

[B45] Gupta D. K. Palma J. M. Corpas F. J. (2018). Antioxidants and antioxidant enzymes in higher plants (Berlin: Springer International Publishing), 1–300.

[B47] Gusain S. Joshi S. Joshi R. (2023). Sensing, signalling, and regulatory mechanism of cold-stress tolerance in plants. Plant Physiol. Biochem. 197, 107646. doi: 10.1016/j.plaphy.2023.107646, PMID: 36958153

[B48] Gusain S. Joshi S. Kumari A. Nath J. Kumari K. Rawat M. . (2025). “ Cold priming and memory induced acquired tolerance and possible mechanism in plants,” in Exogenous Priming and Engineering of Plant Metabolic and Regulatory Genes (USA: Academic Press), 95–106.

[B49] Hasanuzzaman M. Roychowdhury R. Karmakar J. Dey N. Nahar K. Fujita M. (2015). “ Recent advances in biotechnology and genomic approaches for abiotic stress tolerance in crop plants,” in Genomics and proteomics: concepts, technologies and applications ( Apple Academic Press, Burlington), 333–366.

[B50] Hassan M. A. Xiang C. Farooq M. Muhammad N. Yan Z. Hui X. . (2021). Cold stress in wheat: plant acclimation responses and management strategies. Front. Plant Sci. 12, 676884. doi: 10.3389/fpls.2021.676884, PMID: 34305976 PMC8299469

[B51] Hereme R. Galleguillos C. Morales-Navarro S. Molina-Montenegro M. A. (2021). What if the cold days return? Epigenetic mechanisms in plants to cold tolerance. Planta 254, 46. doi: 10.1007/s00425-021-03694-1, PMID: 34370110

[B52] Hoermiller I. I. Ruschhaupt M. Heyer A. G. (2018). Mechanisms of frost resistance in Arabidopsis thaliana. Planta 248, 827–835. doi: 10.1007/s00425-018-2939-1, PMID: 29936546

[B53] Huang B. Fan Y. Cui L. Li C. Guo C. (2022). Cold stress response mechanisms in anther development. Int. J. Mol. Sci. 24, 30. doi: 10.3390/ijms24010030, PMID: 36613473 PMC9820542

[B54] Huang X. Shi H. Hu Z. Liu A. Amombo E. Chen L. . (2017). ABA is involved in regulation of cold stress response in Bermudagrass. Front. Plant Sci. 8, 1613. doi: 10.3389/fpls.2017.01613, PMID: 29081782 PMC5645512

[B55] Hussain M. A. Li S. Gao H. Feng C. Sun P. Sui X. . (2023). Comparative analysis of physiological variations and genetic architecture for cold stress response in soybean germplasm. Front. Plant Sci. 13, 1095335. doi: 10.3389/fpls.2022.1095335, PMID: 36684715 PMC9852849

[B56] Hwarari D. Guan Y. Ahmad B. Movahedi A. Min T. Hao Z. . (2022). ICE-CBF-COR signalling cascade and its regulation in plants responding to cold stress. Int. J. Mol. Sci. 23, 1549. doi: 10.3390/ijms23031549, PMID: 35163471 PMC8835792

[B57] Jahed K. R. Saini A. K. Sherif S. M. (2023). Coping with the cold: unveiling cryoprotectants, molecular signalling pathways, and strategies for cold stress resilience. Front. Plant Sci. 14, 1246093. doi: 10.3389/fpls.2023.1246093, PMID: 37649996 PMC10465183

[B58] Jeon J. Kim J. (2013). Cold stress signalling networks in Arabidopsis. J. Plant Biol. 56, 69–76. doi: 10.1007/s12374-013-0903-y

[B59] Jiao C. Sun J. (2025). SlNAC1 mediates MeSA application-promoted cold resistance by improving transcription of SlNRX2 in tomato fruit. Postharvest Biol. Technol. 223, 113459. doi: 10.1016/j.postharvbio.2025.113459

[B60] Kazan K. (2015). Diverse roles of jasmonates and ethylene in abiotic stress tolerance. Trends Plant Sci. 20, 219–229. doi: 10.1016/j.tplants.2015.02.001, PMID: 25731753

[B61] Kazemi-Shahandashti S. S. Maali-Amiri R. (2018). Global insights of protein responses to cold stress in plants: Signalling, defence, and degradation. J. Plant Physiol. 226, 123–135. doi: 10.1016/j.jplph.2018.03.022, PMID: 29758377

[B62] Kidokoro S. Watanabe K. Ohori T. Moriwaki T. Maruyama K. Mizoi J. . (2015). Soybean DREB 1/CBF-type transcription factors function in heat and drought as well as cold stress-responsive gene expression. Plant J. 81, 505–518. doi: 10.1111/tpj.12746, PMID: 25495120

[B63] Kim J. S. Kidokoro S. Yamaguchi-Shinozaki K. Shinozaki K. (2024). Regulatory networks in plant responses to drought and cold stress. Plant Physiol. 195, 170–189. doi: 10.1093/plphys/kiae105, PMID: 38514098 PMC11060690

[B64] Klatt S. Schinkel C. C. Kirchheimer B. Dullinger S. Hörandl E. (2018). Effects of cold treatments on fitness and mode of reproduction in the diploid and polyploid alpine plant Ranunculus kuepferi (Ranunculaceae). Ann. Bot. 121, 1287–1298. doi: 10.1093/aob/mcy017, PMID: 29462249 PMC6007502

[B65] Kosakivska I. V. Vedenicheva N. P. Babenko L. M. Voytenko L. V. Romanenko K. O. Vasyuk V. A. (2022). Exogenous phytohormones in the regulation of growth and development of cereals under abiotic stresses. Mol. Biol. Rep. 49, 617–628. doi: 10.1007/s11033-021-06802-2, PMID: 34669126

[B66] Lainé C. M. AbdElgawad H. Beemster G. T. (2023). A meta-analysis reveals differential sensitivity of cold stress responses in the maize leaf. Plant Cell Env. 46, 2432–2449. doi: 10.1111/pce.14608, PMID: 37170821

[B67] Levitt J. (2012). Chilling, freezing, and high temperature stresses Vol. 1 (NY, USA: Academic Press).

[B70] Li J. Li Q. Wang F. Ding R. Shang Y. Hu X. . (2025b). Analysis of the SlRAF-like B gene family in tomato and the molecular mechanism of SlRAF7 in regulating cold stress resistance. Plant Sci. 355, 112475. doi: 10.1016/j.plantsci.2025.112475, PMID: 40097049

[B69] Li T. Li B. Wang Y. Xu J. Li W. Chen Z. H. . (2025a). WRKY transcription factors in rice: key regulators orchestrating development and stress resilience. Plant Cell Env. 48, 8388–8406. doi: 10.1111/pce.70124, PMID: 40831341

[B68] Li M. Sui N. A. Lin L. Yang Z. Zhang Y. (2019). Transcriptomic profiling revealed genes involved in response to cold stress in maize. Func. Plant Biol. 46, 830–844. doi: 10.1071/FP19065, PMID: 31217070

[B72] Liao Y. Zou H. F. Wang H. W. Zhang W. K. Ma B. Zhang J. S. . (2008). Soybean GmMYB76, GmMYB92, and GmMYB177 genes confer stress tolerance in transgenic Arabidopsis plants. Cell Res. 18, 1047–1060. doi: 10.1038/cr.2008.280, PMID: 18725908

[B73] Lim C. W. Lee S. C. (2020). ABA-dependent and ABA-independent functions of RCAR5/PYL11 in response to cold stress. Front. Plant Sci. 11, 587620. doi: 10.3389/fpls.2020.587620, PMID: 33101352 PMC7545830

[B74] Liu B. Mo W. J. Zhang D. De Storme N. Geelen D. (2019). Cold influences male reproductive development in plants: a hazard to fertility, but a window for evolution. Plant Cell Physiol. 60, 7–18. doi: 10.1093/pcp/pcy209, PMID: 30602022

[B76] Manasa S. ,. L. Panigrahy M. Panigrahi K. C. Rout G. R. (2022). Overview of cold stress regulation in plants. Botanical. Rev. 88, 359–387. doi: 10.1007/s12229-021-09267-x

[B77] Maric A. (2025). Memories that last: epigenetic regulation of cold stress response prepares plants for subsequent stress events. Plant Physiol. 197, kiae579. doi: 10.1093/plphys/kiae579, PMID: 39471478 PMC11663549

[B78] Mazur R. Gieczewska K. Kowalewska Ł. Kuta A. Proboszcz M. Gruszecki W. I. . (2020). Specific composition of lipid phases allows retaining an optimal thylakoid membrane fluidity in plant response to low-temperature treatment. Front. Plant Sci. 11, 723. doi: 10.3389/fpls.2020.00723, PMID: 32582253 PMC7291772

[B79] Mishra S. Roychowdhury R. Ray S. Hada A. Kumar A. Sarker U. . (2024). Salicylic acid (SA)-mediated plant immunity against biotic stresses: an insight on molecular components and signalling mechanism. Plant Stress 11, 100427. doi: 10.1016/j.stress.2024.100427

[B80] Monteiro-Batista R. D. C. Siqueira J. A. Da Fonseca-Pereira P. Barreto P. Feitosa-Araujo E. Araújo W. L. . (2025). Potential roles of mitochondrial carrier proteins in plant responses to abiotic stress. J. Exp. Bot. 76, 4760–4770. doi: 10.1093/jxb/eraf032, PMID: 39864071

[B81] Moradi L. Siosemardeh A. (2023). Combination of seed priming and nutrient foliar application improved physiological attributes, grain yield, and biofortification of rainfed wheat. Front. Plant Sci. 14, 1287677. doi: 10.3389/fpls.2023.1287677, PMID: 38023831 PMC10644532

[B82] Nakashima K. Ito Y. Yamaguchi-Shinozaki K. (2009). Transcriptional regulatory networks in response to abiotic stresses in Arabidopsis and grasses. Plant Physiol. 149, 88–95. doi: 10.1104/pp.108.129791, PMID: 19126699 PMC2613698

[B83] Nakashima K. Yamaguchi-Shinozaki K. Shinozaki K. (2025). Transcriptional gene network involved in drought stress response: application for crop breeding in the context of climate change. Philos. Trans. B 380, 20240236. doi: 10.1098/rstb.2024.0236, PMID: 40439309 PMC12132078

[B84] Newton A. C. Johnson S. N. Gregory P. J. (2011). Implications of climate change for diseases, crop yields and food security. Euphytica 179, 3–18. doi: 10.1007/s10681-011-0359-4

[B85] Pattnaik D. Dash D. Mishra A. Padhiary A. K. Dey P. Dash G. K. (2021). “ Emerging roles of osmoprotectants in alleviating abiotic stress response under changing climatic conditions,” in Climate impacts on sustainable natural resource management, (UK: John Wiley & Sons Ltd), 303–324.

[B86] Pirzadah T. B. Malik B. Rehman R. U. Hakeem K. R. Qureshi M. I. (2013). “ Signalling in response to cold stress. In Plant signalling: Understanding the molecular crosstalk,” Plant signalling: Understanding the molecular crosstalk ( Springer India, New Delhi), 193–226.

[B87] Qian Z. He L. Li F. (2024). Understanding cold stress response mechanisms in plants: An overview. Front. Plant Sci. 15, 1443317. doi: 10.3389/fpls.2024.1443317, PMID: 39568458 PMC11576170

[B88] Qin F. Sakuma Y. Li J. Liu Q. Li Y. Q. Shinozaki K. . (2004). Cloning and functional analysis of a novel DREB1/CBF transcription factor involved in cold-responsive gene expression in Zea mays L. Plant Cell Physiol. 45, 1042–1052. doi: 10.1093/pcp/pch118, PMID: 15356330

[B89] Ramakrishnan M. Zhang Z. Mullasseri S. Kalendar R. Ahmad Z. Sharma A. . (2022). Epigenetic stress memory: A new approach to study cold and heat stress responses in plants. Front. Plant Sci. 13, 1075279. doi: 10.3389/fpls.2022.1075279, PMID: 36570899 PMC9772030

[B90] Rao M. J. Zheng B. (2025). The role of polyphenols in abiotic stress tolerance and their antioxidant properties to scavenge reactive oxygen species and free radicals. Antioxidants 14, 74. doi: 10.3390/antiox14010074, PMID: 39857408 PMC11761259

[B91] Raza A. Charagh S. Abbas S. Hassan M. U. Saeed F. Haider S. . (2023). Assessment of proline function in higher plants under extreme temperatures. Plant Biol. 25, 379–395. doi: 10.1111/plb.13510, PMID: 36748909

[B92] Raza A. Charagh S. Zahid Z. Mubarik M. S. Javed R. Siddiqui M. H. . (2021). Jasmonic acid: a key frontier in conferring abiotic stress tolerance in plants. Plant Cell Rep. 40, 1513–1541. doi: 10.1007/s00299-020-02614-z, PMID: 33034676

[B93] Ren M. Wang W. Pu S. Shi W. Hu T. Tang Q. . (2022). Assessing the genetic improvement in inbred late rice against chilling stress: consequences for spikelet fertility, pollen viability and anther characteristics. Agronomy 12, 1894. doi: 10.3390/agronomy12081894

[B94] Reyes-Díaz M. Ulloa N. Zuniga-Feest A. Gutiérrez A. Gidekel M. Alberdi M. . (2006). Arabidopsis thaliana avoids freezing by supercooling. J. Exp. Bot. 57, 3687–3696. doi: 10.1093/jxb/erl125, PMID: 16990371

[B95] Ritonga F. N. Chen S. (2020). Physiological and molecular mechanism involved in cold stress tolerance in plants. Plants 9, 560. doi: 10.3390/plants9050560, PMID: 32353940 PMC7284489

[B96] Roychowdhury R. (2014). Crop improvement in the era of climate change (New Delhi, India: IK International Publisher).

[B99] Roychowdhury R. Ballén-Taborda C. Chaturvedi P. (2023b). Characterizing and improving traits for resilient crop development. Front. Plant Sci. 14, 1307327. doi: 10.3389/fpls.2023.1307327, PMID: 37941664 PMC10628715

[B97] Roychowdhury R. Choudhury S. Hasanuzzaman M. Srivastava S. (2020). Sustainable agriculture in the era of climate change (Switzerland: Springer-Nature).

[B103] Roychowdhury R. Das S. P. Das S. Biswas S. Patel M. K. Kumar A. . (2025b). Advancing vegetable genetics with gene editing: a pathway to food security and nutritional resilience in climate-shifted environments. Func. Integrat. Genom. 25, 31. doi: 10.1007/s10142-025-01533-0, PMID: 39891757

[B98] Roychowdhury R. Das S. P. Gupta A. Parihar P. Chandrasekhar K. Sarker U. . (2023a). Multiomics pipeline and omics-integration approach to decipher plant’s abiotic stress tolerance responses. Genes 14, 1281. doi: 10.3390/genes14061281, PMID: 37372461 PMC10298225

[B100] Roychowdhury R. Hada A. Biswas S. Mishra S. Prusty M. R. Das S. P. . (2024a). Jasmonic acid (JA) in plant immune response: unravelling complex molecular mechanisms and networking of defence signalling against pathogens. J. Plant Growth Regul. 44, 89–114. doi: 10.1007/s00344-024-11264-4

[B101] Roychowdhury R. Mishra S. Anand G. Dalal D. Gupta R. Kumar A. . (2024b). Decoding the molecular mechanism underlying salicylic acid (SA)-mediated plant immunity: an integrated overview from its biosynthesis to the mode of action. Physiol. Plantarum. 176, e14399. doi: 10.1111/ppl.14399, PMID: 38894599

[B102] Roychowdhury R. Prasad A. Das S. P. Shah P. Kumar A. Sarker U. . (2025a). “ Metabolomics-assisted breeding: A new omics-integrated avenue for improved stress response in plants,” in High-Throughput Plant Metabolomics (UK: CABI), 292–314.

[B104] Rubio S. Noriega X. Pérez F. J. (2019). Abscisic acid (ABA) and low temperatures synergistically increase the expression of CBF/DREB1 transcription factors and cold-hardiness in grapevine dormant buds. Ann. Bot. 123, 681–689. doi: 10.1093/aob/mcy201, PMID: 30418484 PMC6417478

[B105] Saha P. Debnath B. Sharma N. Dutta S. Barman A. Bera A. . (2025). “ Phytohormones: crucial factors for plant stress tolerance,” in Plant Stress Tolerance (Boca Raton: CRC Press), 113–133.

[B106] Sanghera G. S. Wani S. H. (2008). Innovative approaches to enhance genetic potential of rice for higher productivity under temperate conditions of Kashmir. J. Plant Sci. Res. 24, 99–113.

[B107] Sardoei A. S. Fazeli-Nasab B. (2025). Physiological and molecular mechanisms of freezing in plants. Phyton 94, 1601-1630. doi: 10.32604/phyton.2025.064729

[B108] Satyakam Zinta G. Singh R. K. Kumar R. (2022). Cold adaptation strategies in plants—An emerging role of epigenetics and antifreeze proteins to engineer cold resilient plants. Front. Genet. 13, 909007. doi: 10.3389/fgene.2022.909007, PMID: 36092945 PMC9459425

[B109] Shah S. H. Islam S. Mohammad F. Siddiqui M. H. (2023). Gibberellic acid: a versatile regulator of plant growth, development and stress responses. J. Plant Growth Regul. 42, 7352–7373. doi: 10.1007/s00344-023-11035-7

[B110] Shahan R. (2020). The cold never bothered me anyway: DELLA-interacting growth regulating factors mediate plant growth in cold stress. Plant Cell 32, 797–798. doi: 10.1105/tpc.20.00079, PMID: 32060177 PMC7145467

[B111] Sharma A. Kumar V. Sidhu G. P. S. Kumar R. Kohli S. K. Yadav P. . (2019). “ Abiotic stress management in plants: Role of ethylene,” in Molecular plant abiotic stress: Biology and Biotechnol, (UK: John Wiley & Sons Ltd), 185–208.

[B112] Sharma K. D. Nayyar H. (2016). Regulatory networks in pollen development under cold stress. Front. Plant Sci. 7, 402. doi: 10.3389/fpls.2016.00402, PMID: 27066044 PMC4814731

[B113] Shi Y. Yang S. (2014). “ ABA regulation of the cold stress response in plants,” in Abscisic acid: metabolism, transport and signalling ( Springer Netherlands, Dordrecht), 337–363.

[B114] Shomo Z. D. Li F. Smith C. N. Edmonds S. R. Roston R. L. (2024). From sensing to acclimation: The role of membrane lipid remodelling in plant responses to low temperatures. Plant Physiol. 196, 1737–1757. doi: 10.1093/plphys/kiae382, PMID: 39028871

[B115] Smyth S. J. Webb S. R. Phillips P. W. (2021). The role of public–private partnerships in improving global food security. Global Food Secur. 31, 100588. doi: 10.1016/j.gfs.2021.100588

[B116] Soltani M. Sorahinobar M. Abedi Z. (2022). Comparative Analysis of Expressed sequence tags in Wheat, Rice, and Barley under Cold Stress. Microbiol. Metabol. Biotechnol. 5, 114–127. doi: 10.22104/mmb.2023.6206.1101

[B117] Soualiou S. Duan F. Li X. Zhou W. (2022). Crop production under cold stress: An understanding of plant responses, acclimation processes, and management strategies. Plant Physiol. Biochem. 190, 47–61. doi: 10.1016/j.plaphy.2022.08.024, PMID: 36099808

[B118] Staniak M. Czopek K. Stępień-Warda A. Kocira A. Przybyś M. (2021). Cold stress during flowering alters plant structure, yield and seed quality of different soybean genotypes. Agronomy 11, 2059. doi: 10.3390/agronomy11102059

[B119] Storey J. M. Storey K. B. (2023). Chaperone proteins: universal roles in surviving environmental stress. Cell Stress Chaperones 28, 455–466. doi: 10.1007/s12192-022-01312-x, PMID: 36441380 PMC10469148

[B120] Sukumaran S. Rebetzke G. Mackay I. Bentley A. R. Reynolds M. P. (2022). “ Prebreeding strategies,” in Wheat improvement: Food security in a changing climate ( Springer International Publishing, Cham), 451–469.

[B121] Sun M. Shen Y. Yang J. Cai X. Li H. Zhu Y. . (2020). miR535 negatively regulates cold tolerance in rice. Mol. Breed. 40, 14. doi: 10.1007/s11032-019-1094-0

[B122] Takeno K. (2016). Stress-induced flowering: the third category of flowering response. J. Exp. Bot. 67, 4925–4934. doi: 10.1093/jxb/erw272, PMID: 27382113

[B123] Tarkowski Ł. P. Van Den Ende W. (2015). Cold tolerance triggered by soluble sugars: a multifaceted countermeasure. Front. Plant Sci. 6, 203. doi: 10.3389/fpls.2015.00203, PMID: 25926837 PMC4396355

[B124] Thakur N. R. Ingle K. P. Sargar P. R. Baraskar S. S. Kasanaboina K. Awio B. . (2024). “ Sustainable utilization of wild germplasm resources,” in Sustainable Utilization and Conservation of Plant Genetic Diversity ( Springer Nature Singapore, Singapore), 551–590.

[B125] Theocharis A. Clément C. Barka E. A. (2012). Physiological and molecular changes in plants grown at low temperatures. Planta 235, 1091–1105. doi: 10.1007/s00425-012-1641-y, PMID: 22526498

[B126] Thomashow M. F. (1999). Plant cold acclimation: freezing tolerance genes and regulatory mechanisms. Annu. Rev. Plant Biol. 50, 571–599. doi: 10.1146/annurev.arplant.50.1.571, PMID: 15012220

[B127] Tiwari M. Kumar R. Subramanian S. Doherty C. J. Jagadish S. K. (2023). Auxin–cytokinin interplay shapes root functionality under low-temperature stress. Trends Plant Sci. 28, 447–459. doi: 10.1016/j.tplants.2022.12.004, PMID: 36599768

[B128] Upadhyay R. K. Fatima T. Handa A. K. Mattoo A. K. (2020). Polyamines and their biosynthesis/catabolism genes are differentially modulated in response to heat versus cold stress in tomato leaves (Solanum lycopersicum L.). Cells 9, 1749. doi: 10.3390/cells9081749, PMID: 32707844 PMC7465501

[B129] Vaschetto L. M. (2024). Epigenetics in Crop Improvement: Safeguarding Food Security in an Ever-Changing Climate (Switzerland: Springer Nature).

[B130] Wang F. Liang D. Pei X. Zhang Q. Zhang P. Zhang J. . (2019). Study on the physiological indices of Pinus sibirica and Pinus koraiensis seedlings under cold stress. J. Forestry Res. 30, 1255–1265. doi: 10.1007/s11676-018-0833-0

[B131] Wang J. Song L. Gong X. Xu J. Li M. (2020). Functions of jasmonic acid in plant regulation and response to abiotic stress. Int. J. Mol. Sci. 21, 1446. doi: 10.3390/ijms21041446, PMID: 32093336 PMC7073113

[B132] Wani M. A. Jan N. Qazi H. A. Andrabi K. I. John R. (2018). Cold stress induces biochemical changes, fatty acid profile, antioxidant system and gene expression in Capsella bursa pastoris L. Acta Physiol. Plantar 40, 167. doi: 10.1007/s11738-018-2747-z

[B133] Wei Y. Chen H. Wang L. Zhao Q. Wang D. Zhang T. (2022). Cold acclimation alleviates cold stress-induced PSII inhibition and oxidative damage in tobacco leaves. Plant Signal. Behav. 17, 2013638. doi: 10.1080/15592324.2021.2013638, PMID: 34964430 PMC8920150

[B134] Wingler A. Tijero V. Müller M. Yuan B. Munné-Bosch S. (2020). Interactions between sucrose and jasmonate signalling in the response to cold stress. BMC Plant Biol. 20, 176. doi: 10.1186/s12870-020-02376-6, PMID: 32321430 PMC7178619

[B136] Wu Z. Han S. Zhou H. Tuang Z. K. Wang Y. Jin Y. . (2019). Cold stress activates disease resistance in Arabidopsis thaliana through a salicylic acid dependent pathway. Plant Cell Env. 42, 2645–2663. doi: 10.1111/pce.13579, PMID: 31087367

[B135] Wu J. Nadeem M. Galagedara L. Thomas R. Cheema M. (2022). Recent insights into cell responses to cold stress in plants: Signalling, defence, and potential functions of phosphatidic acid. Env. Exp. Bot. 203, 105068. doi: 10.1016/j.envexpbot.2022.105068

[B138] Xu Y. Fu X. (2022). Reprogramming of plant central metabolism in response to abiotic stresses: A metabolomics view. Int. J. Mol. Sci. 23, 5716. doi: 10.3390/ijms23105716, PMID: 35628526 PMC9143615

[B137] Xu Q. Yan Y. Wei Q. Wang H. Chi C. Pan L. . (2025). Salicylic acid alleviates cold stress in Rice by regulating nutrient absorption, osmotic material content, Antioxidation System, and expression of Cold Tolerance genes. J. Plant Growth Regul. 44, 3240–3272. doi: 10.1007/s00344-024-11615-1

[B139] Yahia N. Wani S. H. Kumar V. (2018). “ CBF-Dependent and CBF-Independent transcriptional regulation of cold stress responses in plants,” in Cold Tolerance in Plants: Physiological, Molecular and Genetic Perspectives ( Springer International Publishing, Cham), 89–102.

[B140] Yu J. Cang J. Lu Q. Fan B. Xu Q. Li W. . (2020). ABA enhanced cold tolerance of wheat ‘dn1’by increasing ROS scavenging system. Plant Signal. Behav. 15, 1780403. doi: 10.1080/15592324.2020.1780403, PMID: 32619128 PMC8570709

[B141] Yu Y. Zhang L. (2023). The wheat NAC transcription factor TaNAC22 enhances cadmium stress tolerance in wheat. Cereal Res. Comm. 51, 867–877. doi: 10.1007/s42976-023-00354-w

[B142] Zhang J. Chen X. Song Y. Gong Z. (2024a). Integrative regulatory mechanisms of stomatal movements under changing climate. J. Integr. Plant Biol. 66, 368–393. doi: 10.1111/jipb.13611, PMID: 38319001

[B144] Zhang R. Yang L. Zhang H. Yang Y. Wen L. Yin A. . (2025). Molecular networks governing plant responses to heat and cold stress. Plants 14, 2073. doi: 10.3390/plants14132073, PMID: 40648082 PMC12252174

[B143] Zhang G. Zhang Y. Kou X. Li J. Luo D. Huang T. . (2024b). Salicylic acid mitigates chilling injury to peaches by improving antioxidant capacity and energy metabolism. Sci. Horticul. 338, 113841. doi: 10.1016/j.scienta.2024.113841

[B146] Zhou X. Muhammad I. Lan H. Xia C. (2022). Recent advances in the analysis of cold tolerance in maize. Front. Plant Sci. 13, 866034. doi: 10.3389/fpls.2022.866034, PMID: 35498657 PMC9039722

[B145] Zhou L. Ullah F. Zou J. Zeng X. (2025). Molecular and physiological responses of plants that enhance cold tolerance. Int. J. Mol. Sci. 26, 1157. doi: 10.3390/ijms26031157, PMID: 39940925 PMC11818088

[B147] Zulfiqar F. Akram N. A. Ashraf M. (2020). Osmoprotection in plants under abiotic stresses: New insights into a classical phenomenon. Planta 251, 3. doi: 10.1007/s00425-019-03293-1, PMID: 31776765

[B148] Zulfiqar F. Ashraf M. Siddique K. H. (2022). Role of glycine betaine in the thermotolerance of plants. Agronomy 12, 276. doi: 10.3390/agronomy12020276

